# Real-world effectiveness and safety of intranasal esketamine for treatment-resistant depression: Data from the enTRD registry

**DOI:** 10.1192/j.eurpsy.2026.12234

**Published:** 2026-06-23

**Authors:** Maximilian Preiss, Valentin Popper, Luna Helene Charlotte Faber, Theresa Baumgartner, Philip Miguel Baberis-Martinez, Edda Winkler-Pjrek, Gregor Gryglewski, Lucie Bartova, Siegfried Kasper, Dan Rujescu-Balcu, Lukas Pezawas

**Affiliations:** 1 Department of Psychiatry and Psychotherapy, Division of General Psychiatry, Medical University of Vienna, Vienna, Austria; 2 Comprehensive Center for Clinical Neurosciences and Mental Health, Medical University of Vienna, Vienna, Austria

**Keywords:** esketamine nasal spray, glutamate, mood disorders, rapid-acting antidepressant, real-world experience, remission, response, treatment-resistant depression

## Abstract

**Background:**

Real-world data for intranasal esketamine in treatment-resistant depression (TRD) are needed to characterize effectiveness, suicidality, and tolerability in routine care. We evaluated induction phase outcomes in outpatients with TRD treated at the University Hospital Vienna.

**Methods:**

This retrospective single-center registry study included adults with TRD initiating supervised intranasal esketamine. The primary endpoint was the change in clinician-rated depressive symptom severity (MADRS) from the pre-treatment (PV) to the end of induction (EOI) visit. MADRS scores across baseline (BL), PV, and EOI were analyzed using a mixed model for repeated measures. Secondary outcomes included MADRS three-factor change, self-rated depression score trajectories (PHQ-9), response/remission rates, suicidal ideation, and tolerability.

**Results:**

Of 229 screened patients, 176 initiated esketamine and 156 completed induction. MADRS at BL was 31.5 (SD 6.4), PHQ-9 was 18.5 (SD 4.7). In the primary model, adjusted mean MADRS was 30.3 at PV and 17.7 at EOI, yielding a change of −12.5 points (95%CI −14.1 to −11.0; *p* < 0.001; Hedges’ *g* = 1.8). All MADRS factors improved, with the largest reduction in the affective–anhedonic domain. PHQ-9 decreased steadily across treatments. Remission was 27.2% and response 57.4% at EOI. Suicidal ideation decreased significantly; no suicide or serious self-harm occurred during induction. Adverse events (AEs) were recorded in 34.7%, with AE-related discontinuation in 3.4%.

**Conclusions:**

Esketamine induction was associated with marked improvement in depressive symptoms and suicidality, with acceptable tolerability under supervised administration. Longer-term registry follow-up and controlled comparative studies are needed to further define the benefit–risk profile beyond induction phase.

## Introduction

Treatment-resistant depression (TRD) is defined in the regulatory setting as failure to achieve a clinically meaningful response despite treatment with at least two antidepressants prescribed at adequate dosage and duration, with confirmed adherence [1–3]. Under this operational definition, TRD affects roughly one-third of patients treated for major depressive disorder (MDD) and is associated with disproportionate chronicity, suicidality, and healthcare and productivity burden [3–6]. In routine clinical care, subsequent treatment steps for TRD typically involve combining antidepressants, augmenting antidepressant therapy with non-antidepressant agents, or switching to an alternative antidepressant [7, 8]. While these strategies reflect optimization within established antidepressant treatment paradigms, intranasal esketamine represents a distinct antidepressant treatment pathway with a novel mechanism of action, approved for use in TRD as an add-on to standard antidepressant therapy [9, 10]. However, the magnitude and clinical relevance of intranasal esketamine’s antidepressant effect in placebo-controlled trials remain debated. While recent systematic reviews, meta-analyses, and re-analyses consistently demonstrate statistically significant symptom reductions, effect sizes are modest, and uncertainty persists as to whether observed benefits translate into clinically meaningful improvement when balanced against adverse events (AEs) and treatment discontinuation [11–14]. Real-world evidence cannot establish causal effectiveness without randomization and a control group, but it is sensitive to heterogeneous patient populations and routine-care contexts, and it can identify implementation patterns, unexpected outcomes, and tolerability issues that guide clinical practice. In this context, real-world evidence captures how intranasal esketamine is delivered in TRD and permits evaluation of symptom change together with discontinuation and safety under routine monitoring. Across published routine-care cohorts from different health systems and practice environments, patterns of dosing, follow-up, and discontinuation vary noticeably, providing pragmatic context for interpreting symptom change beyond protocol-driven randomized controlled trial conditions [15–18].

We therefore conducted a TRD registry study capturing real-world treatment trajectories of patients receiving intranasal esketamine in routine outpatient care. This prespecified induction-phase analysis provides detailed reporting of treatment exposure, concomitant pharmacotherapy, and safety and tolerability outcomes.

## Methods

### Study design and participants

This registry analysis was a monocentric, retrospective, observational evaluation of routinely collected clinical care data from patients with TRD treated with intranasal esketamine (Spravato®) at the TRD outpatient clinic of the Department of Psychiatry and Psychotherapy, University Hospital Vienna (Vienna Healthcare Group). Treatment was initiated on 4 January 2021; data cut-off for the present analysis was 30 November 2025.

In routine care, patients with a referring diagnosis of TRD were evaluated during a pre-induction phase (Figure 1). This standardized baseline work-up comprised confirmation of the referring diagnosis, review of contraindications, psychiatric and somatic comorbidities, and assessment of depressive symptom severity. Adults (≥18 years) deemed clinically suitable were offered initiation of intranasal esketamine. For the induction-phase outcome analyses, the induction analysis population comprised patients who initiated intranasal esketamine at our outpatient clinic (≥1 administration). Within the induction phase, completers were defined as patients who received eight planned administrations, whereas dropouts discontinued treatment before completion.

**Figure 1.**
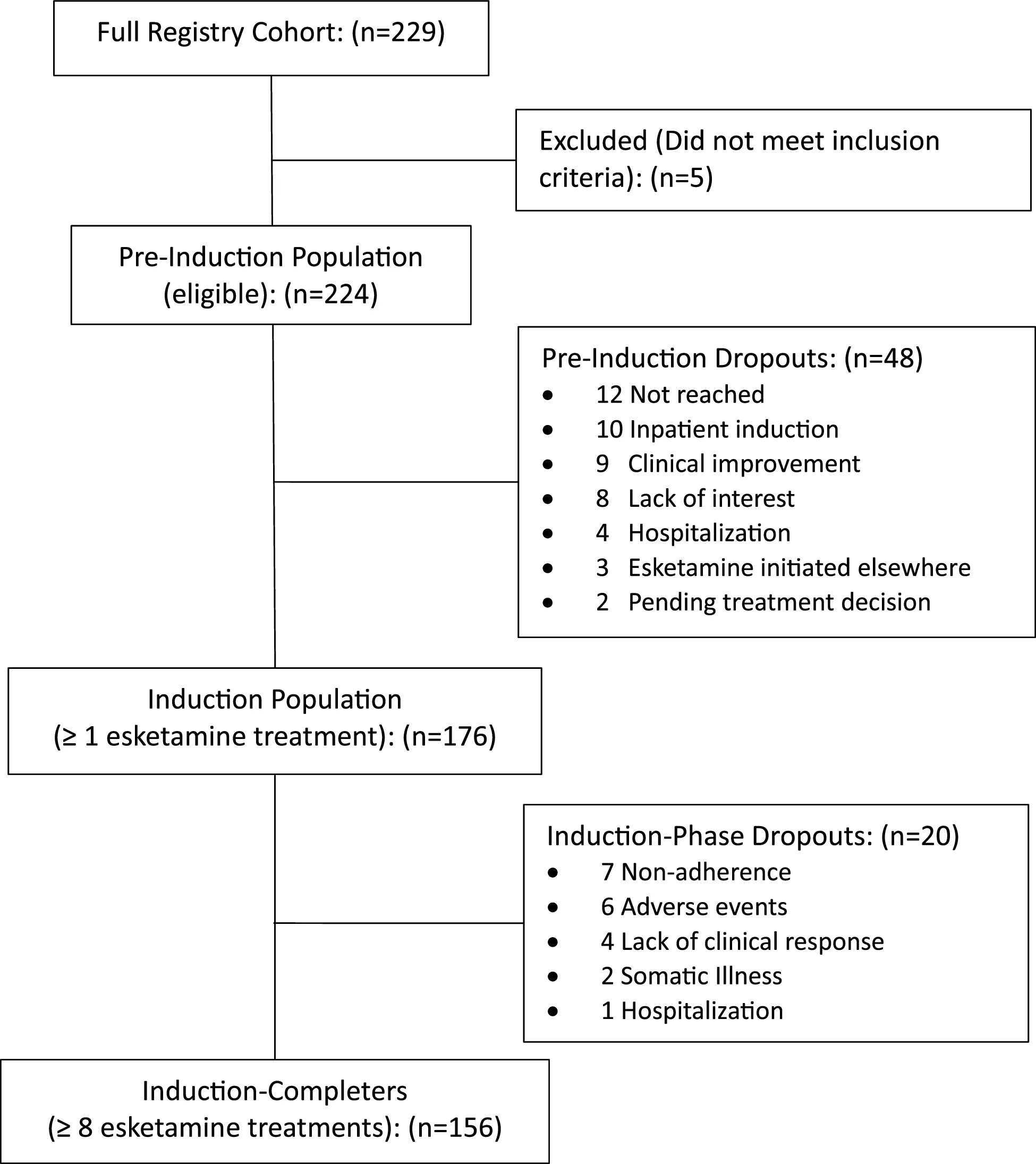
Patient disposition across treatment phases (registry cohort). *Note*: Pre-induction dropouts were defined as patients who did not initiate esketamine treatment after baseline assessment; one primary reason for dropout was assigned per patient. The induction population included all patients who initiated esketamine treatment. Induction-phase dropouts were defined as patients who discontinued treatment during the induction phase (<8 treatments); one primary reason for discontinuation was assigned per patient. Induction completers were defined as patients receiving ≥8 esketamine treatments. Figure 1. long description.

### Treatment, assessments, and outcomes

Treatment took place in the TRD clinic in a low-stimulation group setting. Patients self-administered the nasal spray under the direct supervision of trained staff. During the induction phase, both dosing and administration frequency followed the approved summary of product characteristics, with dosing titrated up to a maximum of 84 mg per administration and treatment scheduled twice weekly for up to eight administrations over 4 weeks. Esketamine was administered in combination with antidepressant pharmacotherapy, typically a selective serotonin reuptake inhibitor (SSRI) or serotonin–noradrenaline reuptake inhibitor (SNRI). Concomitant psychotropic medication was continued or adjusted at the treating clinician’s discretion. Patients were clinically assessed immediately before and after each administration, and discharge was permitted only after post-dose monitoring and clinical stability criteria had been met. During each administration, blood pressure and other vital signs were measured prior to dosing and monitored at 20-minute intervals thereafter. If AE occurred, the treating clinician was notified and provided initial management, including verbal reassurance. If symptoms persisted, symptomatic medication was administered as clinically indicated.

Assessments were conducted as part of routine clinical care and were organized across a total of 11 visits, comprising three predefined visits (baseline visit [BL], pre-treatment visit [PV], and end-of-induction visit [EOI]) and eight treatment visits (V1–V8). At BL, patients underwent a structured clinical evaluation to confirm TRD, assess contraindications, and exclude pseudo-resistance. Descriptive variables were recorded, including sociodemographic data, clinical history, comorbidity, and prior treatment history. Current psychopharmacotherapy was reviewed. Where the baseline work-up indicated insufficient prior antidepressant exposure or pseudo-resistance, pharmacotherapy was adjusted and patients were re-evaluated before esketamine initiation. At PV, clinician-rated depressive symptom severity was assessed prior to the first intranasal esketamine administration. Patient-reported symptoms and side effects were collected at each treatment visit. At EOI, clinician-rated outcomes were reassessed and continuation into maintenance was decided.

Outcomes were defined prior to database lock in a statistical analysis plan (SAP) (see Supplementary Appendix). The primary effectiveness outcome was the change in the Montgomery–Åsberg Depression Rating Scale (MADRS) total score from PV to EOI. Secondary endpoints were prespecified as follows: Secondary endpoints examined (i) change in a predefined three-factor MADRS structure and factor-specific improvements [19] including the prognostic value of the hopelessness factor [20], (ii) the longitudinal course of self-rated depressive symptom severity and whether symptom change differed between early (V1–V4) and late (V4–V8) induction phases, (iii) MADRS-based response (≥50% reduction) and remission (MADRS ≤10) at PV and EOI relative to BL, and (iv) safety, tolerability, and suicidality outcomes.

### Statistical analysis

All analyses were conducted in R (version 4.2) and were prespecified in the SAP prior to database lock. Analyses were performed in the pre-induction cohort (*n* = 224) and the predefined induction analysis population (*n* = 176). Continuous variables were summarized with mean (SD) or median (IQR), and categorical variables with *n* (%). All tests are two-sided with *α* = 0.05. The primary endpoint was prioritized for confirmatory interpretation, whereas secondary and exploratory endpoints were interpreted descriptively without adjustment for multiplicity. Effect sizes and 95% confidence intervals (CI) were emphasized.

Primary analysis: MADRS total scores across all three main timepoints (BL, PV, and EOI) were analyzed using a likelihood-based mixed model for repeated measures (MMRM), with visit specified as a categorical fixed effect and with an unstructured within-patient covariance matrix. Age and sex were included as time-invariant covariates because they are clinically relevant and fully observed [21, 22]. Within-patient correlation was modeled using an unstructured covariance matrix with visit-specific residual variances. The primary estimand was the adjusted mean difference between PV and EOI, derived from model-based least-squares means. Likelihood-based MMRM provides valid inference under a missing-at-random (MAR) assumption and efficiently uses all available repeated measurements without requiring outcome imputation as a primary strategy [23, 24]. Sensitivity analyses: Robustness was examined using a covariate-adjusted change-score model (ANCOVA) including baseline MADRS at PV, age, sex, and Patient Health Questionnaire-9 (PHQ-9) at PV. Furthermore, multiple imputation of MADRS values was applied at BL, PV, and EOI under MAR using chained equations (MICE), followed by re-fitting the primary MMRM within each imputed dataset and Rubin pooling. Missing-not-at-random (MNAR) departures were explored using delta-adjusted pattern-mixture (tipping-point) analyses [25, 26]. This hierarchy follows contemporary guidance for longitudinal clinical data [23–26].

Secondary analyses: MADRS factor scores were analyzed using mixed models that jointly modeled factor and visit effects (including factor-by-visit interaction) to estimate factor-specific PV-EOI contrasts and between-factor differences [19]. The prognostic value of baseline hopelessness for non-response at EOI was evaluated using multivariable logistic regression adjusted for baseline MADRS severity, age, and sex. PHQ-9 symptom course across induction sessions (V1–V8) was analyzed using a piecewise MMRM with an early (V1–V4) and late (V4–V8) slope, adjusted for age and sex; the slope difference was tested via a Wald test on the late-phase component.

Effect sizes were reported as Hedges’ *g*, calculated from model-estimated adjusted changes divided by the standard deviation at the reference timepoint (PV for MADRS; V1 for PHQ-9), with small-sample correction [27, 28]. Response and remission rates from BL to PV and EOI, respectively, were summarized descriptively and then compared using a paired proportions test (McNemar’s test). Safety and suicidality outcomes were summarized descriptively, without imputation.

## Results

### Patient flow

During the study period, 229 referred patients underwent the initial TRD assessment (Figure 1). Five patients were excluded as ineligible, yielding a pre-induction cohort of 224 patients. Of these, 176 patients initiated intranasal esketamine treatment at our TRD clinic and constituted the induction analysis population used for effectiveness and safety analyses. Forty-eight eligible patients did not initiate esketamine and were not included in induction outcome analyses. Reasons included inability to contact the patient, esketamine induction outside the outpatient pathway, and clinical improvement before initiation. Of those initiating treatment, 156 patients completed the induction phase and 20 patients discontinued before completion. Patients who discontinued contributed all available data up to the point of discontinuation.

### Baseline characteristics and treatment context

In the induction analysis population (*n* = 176), mean age was 40.8 (SD 12.7) years and 54.0% of patients were female (Table 1; for characteristics of pre-induction cohort, see Supplementary Appendix). Mean age at onset of depression was 22.7 (SD 11.7) years. Baseline symptom severity was substantial, with a mean MADRS total score of 31.5 (SD 6.4) and a mean PHQ-9 total score of 18.5 (SD 4.7). Median Clinical Global Impression–Severity at BL was 5 (IQR 5–6). The predominant ICD-10 diagnoses were moderate (F33.1; 47.7%) and severe (F33.2; 45.5%) depressive episodes of recurrent depressive disorder, and 74.9% fulfilled criteria for chronic MDD. Prior neurostimulation was uncommon. Psychiatric comorbidity was recorded in 79.5%, most commonly anxiety disorders (65.9%); a past-year substance use disorder was documented in 24.6%. A history of hypomanic/manic episodes was documented in 9.9%. Somatic comorbidity was present in 51.1%, including hypertension (15.3%) and hypothyroidism (13.1%). Patients exhibited marked treatment resistance, with a median of 5 (IQR 3–7) prior antidepressant trials spanning a median of 4 (IQR 3–5) antidepressant classes, and 1 (IQR 0–2) adequate augmentation trial. At BL, 83.1% were receiving an SSRI or SNRI and pharmacotherapy was commonly optimized, with regimen changes in 67.6%. The most frequent categories were antidepressant initiation (21.0%), combined antidepressant and augmentation regimen change (17.0%), and antidepressant dose adjustment (9.7%). After adjustment at BL, concomitant antidepressants most frequently included venlafaxine (50.0%), mirtazapine (35.8%), bupropion (21.6%), trazodone (19.9%), and duloxetine (14.2%). Augmentation strategies commonly involved quetiapine (28.4%) and aripiprazole (24.4%), with lamotrigine (19.3%) and lithium (6.8%) used less frequently (Table 2).

**Table 1.** Baseline characteristics of induction analysis cohort Table 1. long description.

Variable	Induction population	Induction completers	Induction dropouts
Patients (*n*)	176	156	20
Age (years)	40.8 (12.7)	40.8 (12.8)	40.6 (12.3)
Sex (female)	95 (54%)	88 (56.4%)	7 (35%)
Diagnosis (ICD–10)			
Bipolar disorder (F31.x)	7 (4%)	6 (3.8%)	1 (5%)
Depressive episode (F32.x)	4 (2.3%)	4 (2.6%)	0 (0%)
Recurrent depression (F33.x)	165 (93.8%)	146 (93.6%)	19 (95%)
Depression severity			
Moderate	88 (50.9%)	78 (51%)	10 (50%)
Severe	85 (49.1%)	75 (49%)	10 (50%)
Age at depression onset (years)	22.7 (11.7)	22.7 (12.1)	22.6 (8.6)
Current episode duration (months)	12 [6; 24]	12 [6; 23.2]	17.5 [9; 29.5]
MADRS total score	31.5 (6.4)	31.7 (6.5)	30.2 (6.1)
PHQ–9 total score	18.5 (4.7)	18.6 (4.7)	17.5 (4.7)
CGI-S	5 [5; 6]	5 [5; 6]	5 [5; 6]
Number of adequate AD trials	5 [3; 7]	5 [3; 7]	5.5 [4; 6.2]
Prior ECT	8 (4.6%)	6 (3.9%)	2 (10.5%)
Prior esketamine exposure	19 (11.1%)	16 (10.5%)	3 (16.7%)
Psychiatric comorbidity	140 (79.5%)	124 (79.5%)	16 (80%)
Substance use disorder (past year)	43 (24.6%)	35 (22.6%)	8 (40%)
Anxiety disorder in the past year	116 (65.9%)	106 (67.9%)	10 (50%)
Current suicidal ideation	26 (14.8%)	26 (16.7%)	0 (0%)
Suicide attempt ever	35 (21.7%)	33 (23.1%)	2 (11.1%)
Somatic comorbidity	90 (51.1%)	78 (50%)	12 (60%)

*Note: Baseline characteristics of the induction analysis population (≥1 intranasal esketamine administration), induction completers (8 administrations), and induction dropouts (<8 administrations). Data are presented as mean (SD), median [IQR], or n (%), as appropriate. Denominators may vary because of missing data.*

Abbreviations: MADRS, Montgomery-Åsberg Depression rating scale; PHQ-9, Patient Health Questionnaire-9; CGI-S, Clinical Global Impression-Severity; AD, antidepressant; ECT, electroconvulsive therapy.

**Table 2.** Medication change before and after baseline Table 2. long description.

Class	Drug	Before baseline		After baseline	
		*n* (%)	mg/day, mean (SD)	*n* (%)	mg/day, mean (SD)
SSRI	Escitalopram	13 (7.4%)	15.4 (5.2)	18 (10.2%)	16.4 (6.4)
SSRI	Paroxetine	4 (2.3%)	–	6 (3.4%)	33.3 (10.3)
SSRI	Sertraline	12 (6.8%)	141.7 (101.9)	15 (8.5%)	150 (88.6)
SNRI	Duloxetine	22 (12.5%)	96.8 (34.6)	25 (14.2%)	98.4 (33)
SNRI	Milnacipran	15 (8.5%)	110 (33.8)	13 (7.4%)	101.9 (49.4)
SNRI	Venlafaxine	55 (31.2%)	234.5 (100.3)	88 (50%)	258.1 (75.3)
NDRI	Bupropion	41 (23.3%)	307.3 (105.8)	38 (21.6%)	323.7 (89.1)
NaSSA	Mirtazapine	38 (21.6%)	38.8 (15.4)	63 (35.8%)	41.4 (18)
SMS	Vortioxetine	14 (8%)	27.1 (35.6)	15 (8.5%)	15.7 (5.9)
SARI	Trazodone	38 (21.6%)	125.7 (61.6)	35 (19.9%)	138.6 (79.4)
MAOI	Moclobemide	3 (1.7%)	–	5 (2.8%)	330 (67.1)
MS	Lamotrigine	27 (15.3%)	224.1 (132.7)	34 (19.3%)	202.9 (127)
	Lithium	10 (5.7%)	922.5 (326.1)	12 (6.8%)	768.8 (377.2)
AAP	Aripiprazole	31 (17.6%)	9.1 (6.8)	43 (24.4%)	7.9 (5.1)
	Quetiapine	41 (23.3%)	199.1 (161.4)	50 (28.4%)	180.5 (153.2)
Other	Levothyroxine	11 (6.2%)	51.1 (49.4)	10 (5.7%)	52.5 (41.6)

*Note:* Selection of medication changes from pre-baseline to post-baseline assessment in the induction population (≥1 intranasal esketamine administration). For each drug, n (%) receiving the medication and mean daily dose (mg/day, mean [SD]) are reported. Mean dose is shown only when n > 4 among users of that drug. Drugs are grouped by class. Full medication listing is provided in the Supplementary Appendix.

Abbreviations: SSRI, selective serotonin reuptake inhibitor; SNRI, serotonin–norepinephrine reuptake inhibitor; NDRI, norepinephrine–dopamine reuptake inhibitor; NaSSA, noradrenergic and specific serotonergic antidepressant; SMS, serotonin modulator and stimulator; SARI, serotonin antagonist and reuptake inhibitor; MAOI, monoamine oxidase inhibitor; AAP, atypical antipsychotic; MS, mood stabilizer; Other, other/uncategorized.

Baseline characteristics were comparable between the pre-induction cohort and the induction analysis population, with no clinically meaningful differences (see Supplementary Appendix). Compared with completers, induction dropouts tended to have longer episode duration and higher rates of past-year substance use disorder and prior electroconvulsive therapy trials.

### Intranasal esketamine exposure

In the induction analysis population, the median number of intranasal esketamine administrations was 8 (IQR 4–8), cumulative average dosing per induction treatment was 462 mg (SD 257), with an average of 76 mg per session. The median induction treatment interval was 30 days (IQR 28.8–37). The median pre-induction interval was 96 days (IQR 43–183), with the total interval at 125.5 days (IQR 72–207).

### Symptom outcomes

In the primary MMRM analysis of MADRS total scores, adjusted mean values were 31.7 (95%CI 30.6–32.7) at BL and 30.3 (95%CI 29.2–31.3) at PV, indicating minimal change prior to esketamine treatment initiation (Figure 2). By EOI, adjusted mean MADRS decreased to 17.7 (95%CI 16.2–19.3). The primary estimand (EOI − PV) was −12.5 points (95%CI −14.1 to −11.0; *p* < 0.001), corresponding to a Hedges’ *g* of 1.8 (Table 3). Findings were robust in prespecified sensitivity analyses. ANCOVA estimated an adjusted PV-to-EOI change of −12.3 points (95%CI −13.7 to −10.8; *p* < 0.001). Multiple imputation yielded −12.6 MADRS points (95%CI −14.2 to −11.1; *p* < 0.001), and delta-adjusted tipping-point analyses attenuated the estimate only moderately from −12.6 to −11.7, remaining highly significant throughout (*p* < 0.001). In secondary endpoint set (A), all factor scores improved from PV to EOI (Figure 3). Adjusted means decreased from 3.6 to 2.0 for the affective–anhedonic factor, from 2.7 to 1.6 for the anxiety–vegetative factor, and from 2.4 to 1.3 for the hopelessness factor. Improvements were greater for the affective–anhedonic factor than for the anxiety–vegetative factor (between-factor difference in change −0.6, 95%CI −0.7 to −0.4; *p* < 0.001) and the hopelessness factor (−0.5, 95%CI −0.7 to −0.3; *p* < 0.001). In the exploratory model linking hopelessness with overall MADRS improvement, the association did not reach statistical significance (OR per 1 SD = 1.5, 95%CI 0.9–2.5; *p* = 0.091). In secondary outcome set (B), PHQ-9 decreased during both the early phase and the late phase, with a total V1–V8 estimand of −4.9 points (95%CI −5.7 to −4.2; *p* < 0.001) and Hedges’ *g* of 1.0 (Figure 4). There was no evidence for a different rate of change between phases (late-early difference −0.1, 95%CI −0.4 to 0.1; *p* = 0.373) (see Supplementary Appendix for outcome details).

**Figure 2.**
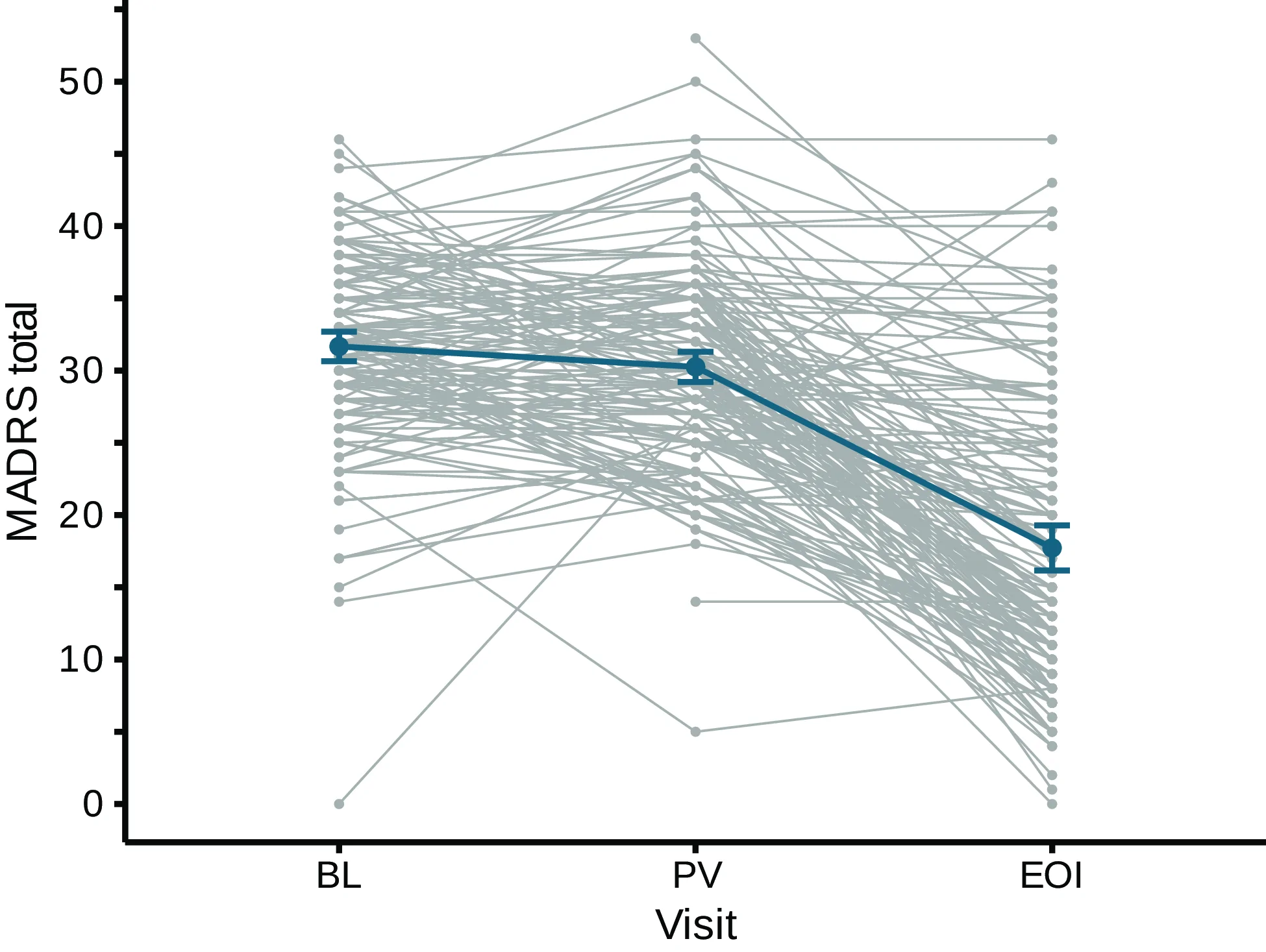
Adjusted MADRS total score over induction: least-squares means from baseline to end of induction. *Note:* Adjusted MADRS total scores from baseline to end of induction. Blue points represent model-based least-squares means estimated from the primary MMRM, including baseline (BL), pre-treatment visit (PV), and end of induction (EOI), adjusted for age and sex with a patient-level random intercept and unstructured covariance. Error bars indicate 95% confidence intervals. Gray points with lines represent patient-level trajectories. The outlier trajectory with MADRS = 0 at BL reflects a patient with seasonal affective disorder who entered assessment in remission and relapsed during winter prior to starting esketamine. Figure 2. long description.

**Table 3.** Core effectiveness outcomes (induction analysis population) Table 3. long description.

Outcome	Model/estimand	Estimand	95% CI	*P* value	Hedges’ *g*
MADRS total	MMRM adjusted mean at BL	31.7	30.6 to 32.7	–	–
	MMRM adjusted mean at PV	30.3	29.2 to 31.3	–	–
	MMRM adjusted mean at EOI	17.7	16.2 to 19.3	–	–
	Primary estimand: EOI − PV	−12.5	−14.1 to −11.0	<0.001	1.8
PHQ–9 total	Piecewise mixed model: early slope (V1–V4)	−0.6	−0.8 to −0.5	<0.001	–
	Piecewise mixed model: late slope (V4–V8)	−0.8	−0.9 to −0.6	<0.001	–
	Late–early slope difference	−0.1	−0.4 to 0.1	0.373	–
	Secondary estimand: V8–V1	−4.9	−5.7 to −4.2	<0.001	1.0

*Note:* MADRS estimates are model-based least-squares means from likelihood-based linear mixed-effects model (MMRM) including fixed effects for time (baseline, pre-treatment visit [PV], end of induction [EOI]) and covariates (age, sex), with within-subject correlation accounted for by the repeated-measures structure. Hedges’ *g* was calculated as the adjusted PV→EOI mean change divided by the PV MADRS SD, applying the small-sample correction. Negative values indicate symptom improvement.

*Abbreviations: MADRS, Montgomery–Åsberg Depression rating scale; PHQ-9, Patient Health Questionnaire-9.*

**Figure 3.**
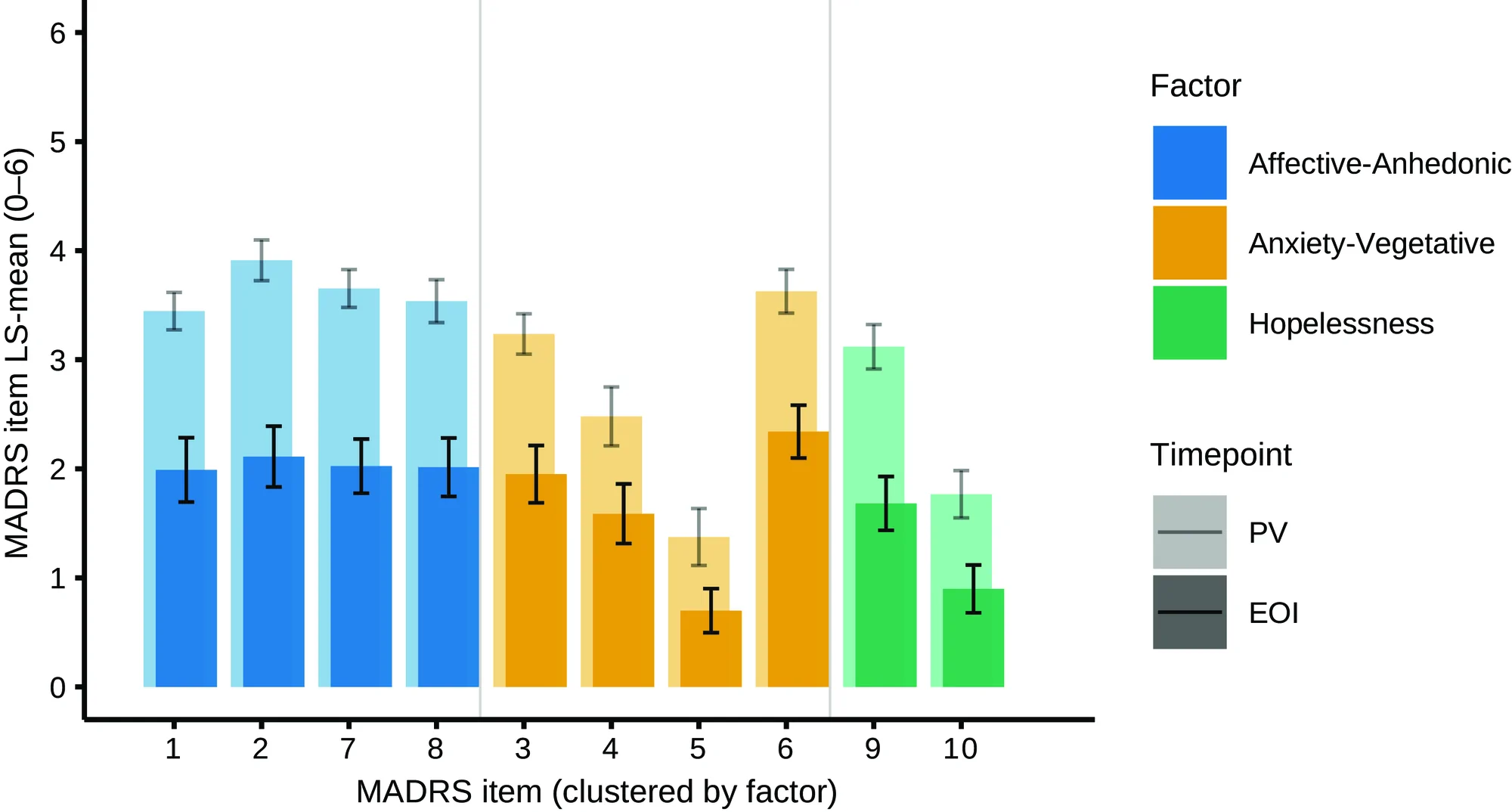
Adjusted MADRS factor scores during induction: least-squares means by symptom cluster. *Note:* Model-based least-squares means for the three MADRS factor clusters (affective–anhedonic, anxiety–vegetative, and hopelessness) from pre-induction visit (PV) to end-of-induction (EOI). Factor scores were calculated as the mean of the items comprising each cluster. Estimates were obtained from the prespecified mixed model framework used for longitudinal analyses. Error bars indicate 95% confidence intervals. Differences in the magnitude of change across clusters illustrate domain-specific contributions to overall MADRS improvement. Figure 3. long description.

**Figure 4.**
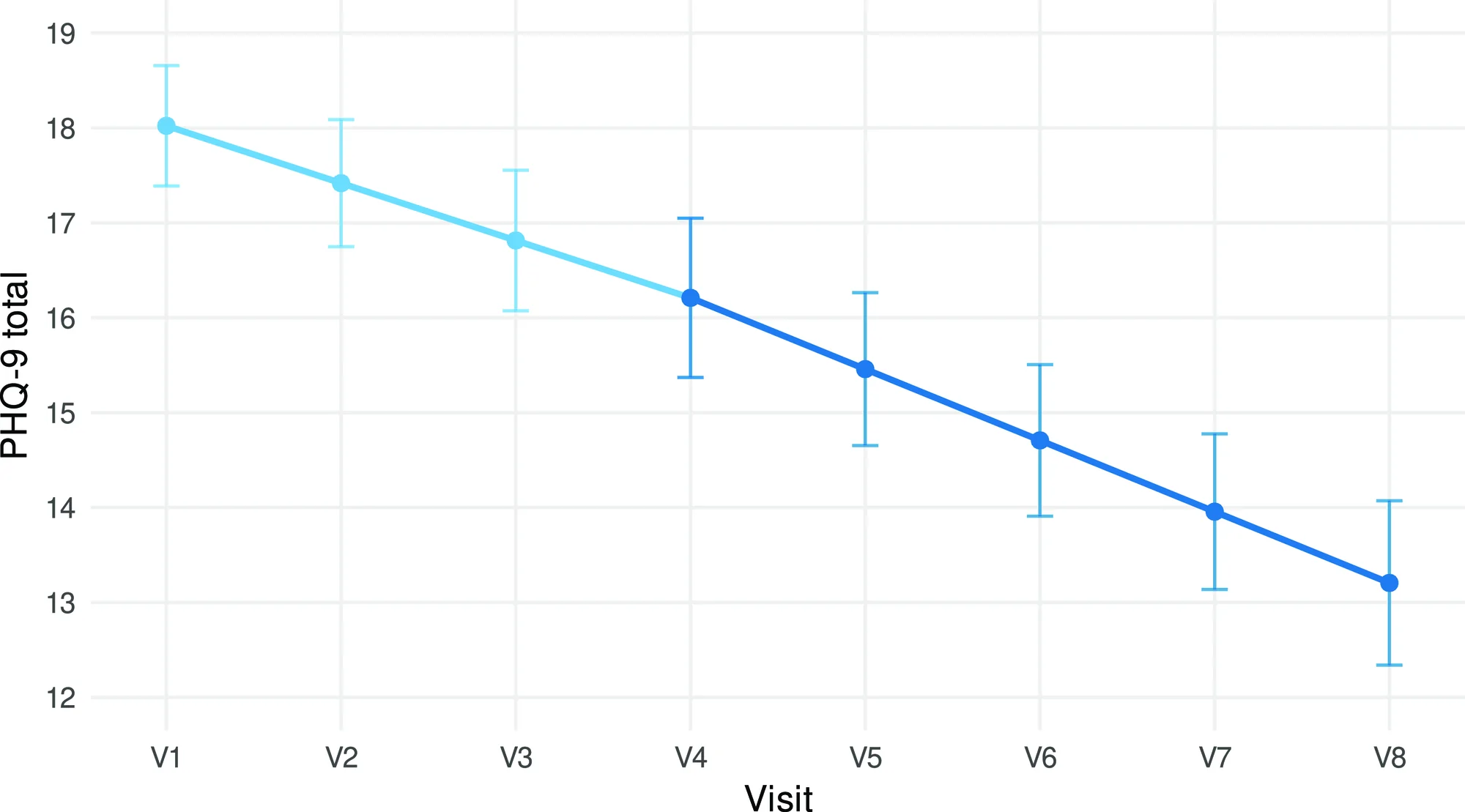
Adjusted PHQ-9 total score over induction: least-squares means across treatment sessions. *Note:* Adjusted PHQ-9 total scores across induction sessions. Points represent model-based least-squares means estimated from the prespecified piecewise mixed model across treatment sessions V1–V8. Error bars indicate 95% confidence intervals. Color shades of lines indicate slopes of treatment phases that were compared in statistical analyses. Figure 4. long description.

### Response and remission

In available-case analyses, remission at PV was rare (1%; *n* = 1/166) and response ≥50% from BL to PV was uncommon (1%; *n* = 2/144); most patients showed no response (88%; *n* = 127/144) (Table 4). By EOI, remission was observed in 27.2% (*n* = 43/158) and response ≥50% in 57.4% (*n* = 78/136). In paired PV–EOI data (*N* = 133), both remission and response ≥50% increased significantly (McNemar *p* < 0.001 each). In conservative sensitivity analyses using last observation carried forward imputation (missing outcomes counted as non-response), estimates were attenuated (EOI remission: 24.4%; response ≥50%: 44.3%) but remained clinically relevant (see Supplementary Appendix).

**Table 4.** MADRS response and remission outcomes Table 4. long description.

Outcome	Time point(*s*)	Patient (*n*)	Events (*n*)	Estimate	95% CI	*P* value
Remission	PV	166	1	0.6%	0.0 to 3.3	–
	EOI	158	43	27.2%	20.4 to 34.9	–
	PV vs EOI (paired)	133	–	–	–	<0.001
Response	PV	144	2	1.4%	0.2 to 4.9	–
	EOI	136	78	57.4%	48.6 to 65.8	–
	PV vs EOI (paired)	133	–	–	–	<0.001

*Note:* Percentages calculated using denominator in “Patient (*n*)” (i.e., patients with MADRS at BL and respective time point). “Events” is number meeting the criterion. Paired PV vs EOI comparisons performed with McNemar’s test among patients with MADRS available at BL, PV, and EOI. Response, MADRS ≥ 50% reduction from BL; Remission, MADRS ≤ 10 total score.

### Suicidality and safety

Markers of suicidality were frequent at BL, with 14.8% reporting current suicidal ideation, 40.9% a history of severe suicidality, and 21.7% at least one prior suicide attempt. Suicidal ideation, as measured by the suicidal ideation item 10 of the MADRS, decreased from PV to EOI, with an adjusted mean change of −0.87 points (95%CI −1.10 to −0.63; *p* < 0.001). PHQ-9 item 9 likewise decreased across induction from V1 to V8, with an adjusted mean change of −0.38 points (95%CI −0.52 to −0.25; *p* < 0.001) (see Supplementary Appendix).

During the induction phase, at least one AE was recorded in 34.7% of patients (95%CI 27.7–42.2). The most frequently documented categories were other AEs like nausea and vomiting (22.2%, 95%CI 16.3–29.0) and significant blood pressure elevation (10.2%, 95%CI 6.2–15.7) (Table 5). Clinically relevant dissociation was documented in 9.7% (95%CI 5.7–15.0) and sedation in 3.4% (95%CI 1.3–7.3). On a per-administration basis, AEs were documented in 7.8% of administrations (95%CI 6.5–9.3). Clinically relevant dissociation occurred in 2.0% (95%CI 1.3–2.9), sedation in 0.8% (95%CI 0.4–1.4), and blood pressure–related events in 1.4% (95%CI 0.9–2.2) of administrations. Treatment discontinuation attributed to AEs occurred in 3.4% of patients (*n* = 6). Reported reasons for discontinuation included nausea and vomiting, dissociation with hallucinations, and, in one case, triggering of a migraine attack. One patient required hospital admission because of a marked worsening of depressive symptoms. No events of suicide or serious self-harm were recorded in the induction population.

**Table 5.** Safety and tolerability during induction Table 5. long description.

Safety outcome	Patient-level incidence	95% CI	Session-level incidence	95% CI
Any AE	34.7%	27.7 to 42.2	7.8%	6.5 to 9.3
Blood-pressure–related events	10.2%	6.2 to 15.7	1.4%	0.9 to 2.2
Dissociation	9.7%	5.7 to 15.0	2.0%	1.3 to 2.9
Sedation	3.4%	1.3 to 7.3	0.8%	0.4 to 1.4
Other AE	22.2%	16.3 to 29.0	5.5%	4.4 to 6.9
Discontinuation due to AE	3.4%	1.3 to 7.3	–	–

*Note: Patient-level incidence denotes the proportion of patients with ≥1 event during induction. Session-level incidence denotes the proportion of induction administrations with an event recorded, calculated across all administrations delivered during induction.*

Abbreviations: AE, adverse event.

## Discussion

### Principal findings

In this monocentric real-world registry study of patients with TRD, intranasal esketamine induction was associated with a marked and clinically meaningful reduction in depressive symptom severity. MADRS total scores decreased by an adjusted mean of 12.5 points from pre-treatment to EOI, corresponding to a large effect size (Hedges’ *g* = 1.8). Symptom levels remained largely stable between BL and PV, as expected in TRD cohorts with persistently high symptom burden [8], supporting the attribution of subsequent observed changes to esketamine exposure rather than to non-specific effects or regression to the mean. This improvement was supported by consistent findings across prespecified sensitivity analyses, indicating robustness to plausible departures from MAR assumptions.

Factor-analytic outcomes revealed differential improvement across depressive symptom domains: all three MADRS factors improved significantly, but the affective–anhedonic domain showed the most pronounced change relative to the anxiety–vegetative and hopelessness domains. Patient-reported PHQ-9 scores decreased steadily across induction sessions, with significant slopes in both early and late phases and no evidence of a plateau (*p* = 0.373 for between-phase difference). By EOI, 57% of patients with available data achieved response and 27% achieved remission, rates that remained meaningful under non-responder imputation. Suicidal ideation improved significantly on both MADRS item 10 and PHQ-9 item 9.

### Comparison with previous esketamine and TRD studies

The effect size in our cohort is consistent with the pooled standardized mean change reported in a recent meta-analysis of nine real-world esketamine studies (Hedges’ *g* = −1.98, 95%CI −2.94 to −1.03; *p* < 0.001) [21], supporting the external consistency of our induction-phase estimate. In that meta-analysis, treatment effectiveness was not significantly influenced by comorbid anxiety, personality, or substance use disorders, a finding that aligns with the clinical profile of our cohort. Notably, clinical complexity in our cohort was high: 74.9% fulfilled criteria for chronic MDD and psychiatric comorbidity was present in 79.5%, including past-year substance use disorder in 24.6%. This comorbidity burden appears higher than in most European routine-care cohorts [15–18]. Response and remission rates, however, are broadly comparable: Martinotti et al. reported 64% response and 41% remission in the Italian REAL-ESK study [17], Conejero et al. observed remission rates of approximately 55% in a European multicenter cohort [18], Samalin et al. reported 40% response and 20% remission after 1 month in the French ESKALE cohort [16], and Brendle et al. documented continuous PHQ-9 improvement across sessions in a US retrospective study [15]. Many studies capture outcomes at coarse intervals and therefore cannot resolve session-by-session dynamics [16, 17]. By adding repeated PHQ-9 assessments across administrations, we extend this literature by demonstrating a steady, continuous improvement signal during induction. We applied the validated three-factor MADRS model described by Borentain et al. [19] to a routine-care cohort and found that, similar to the trial setting, improvement occurred across all factors, with the affective–anhedonic domain most prominently driving overall change. Our cohort, unlike the trial sample, included substantial baseline suicidality, and we observed concurrent improvement in both suicidal ideation indicators and the hopelessness domain.

In the prespecified exploratory model linking baseline hopelessness with overall MADRS improvement, the association did not reach statistical significance. This likely reflects limited statistical power due to few non-response events and additional loss of information under complete case analysis, and may also indicate attenuation from adjustment for baseline MADRS severity, which partially incorporates hopelessness items, introducing collinearity.

The safety profile during the induction phase was consistent with supervised outpatient esketamine administration and with the AE prevalence in recent meta-analytical evidence of de Oliveira Lapa et al. [21] (82%, 95%CI 69–92). In our cohort, AEs occurred in 34.7% of patients per patient and 7.8% per session, with dissociation (9.7%), blood-pressure-related events (10.2%), and sedation (3.4%) as the leading categories. The majority were mild and transient, with only rare need for extended observation. Induction-phase dropout was 11.4%, with 3.4% discontinuing due to AEs, broadly comparable to discontinuation rates reported in European real-world esketamine cohorts [16, 17]. No events of suicide or serious self-harm were recorded during the induction phase.

### Strengths and limitations

Strengths include the use of a prespecified SAP with a MMRM and multiple sensitivity analyses (ANCOVA, MICE, and MNAR tipping-point approaches), strengthening robustness in the presence of missing data. The comparatively large cohort reflects routine outpatient TRD care with substantial baseline illness severity, psychiatric and somatic comorbidity, and polypharmacy, supporting external validity for similar tertiary settings. Outcome assessment combined clinician-rated MADRS at key visits with repeated patient-reported PHQ-9 across administrations, enabling combined interpretation of symptom change; any imperfect, nonlinear correspondence between MADRS and PHQ-9 in our real-world data is expected, given that this pattern is also observed under controlled trial conditions [29]. We report treatment context in detail, including time intervals, exposure, medication optimization prior to initiation, and concomitant treatments, alongside a transparent patient-flow diagram distinguishing pre-induction attrition from induction discontinuation.

Limitations relate primarily to the retrospective, single-center, observational design without a parallel comparator group, which precludes causal inference and limits attribution of change specifically to esketamine versus time, contact intensity, expectancy, and concomitant treatment adaptations. Selection and survivor bias are possible because non-initiators and early discontinuers may differ systematically from completers. AEs were captured from routine documentation rather than structured rating scales, which may underestimate event frequency. The focus on the induction phase restricts inference on maintenance outcomes and durability; notably, one meta-analysis found that maintenance-phase effects were significantly larger than induction-phase effects [21], underscoring the importance of longer follow-up. Finally, despite robust handling of missing outcome data, residual confounding from unmeasured clinical factors and changes in concomitant treatments remains possible.

### Clinical and research implications

In a specialized outpatient setting with routine monitoring, our findings support intranasal esketamine induction as a feasible care pathway for severe, highly comorbid TRD, with clinically meaningful improvement in depressive symptoms and suicidality. Given ongoing debate about the clinical relevance of average effects in placebo-controlled trials [11, 12, 14], real-world data remain essential to contextualize benefit–risk ratio in the polypharmacy and adherence constraints of routine practice. Our separation of pre-induction non-initiation from induction discontinuation may help services interpret real-world effectiveness and plan access pathways, as discontinuation and follow-up patterns vary substantially across routine-care cohorts [18, 21]. Future work will prioritize longer-term registry follow-up to characterize maintenance trajectories, persistence, and discontinuation reasons. Comparative designs and more standardized reporting of concomitant treatment adaptations and AE ascertainment are needed to reduce heterogeneity across settings. Domain-level outcomes (MADRS factors) and repeated patient-reported measures could be leveraged to identify predictors of response, remission, and discontinuation and to inform personalized care pathways [20].

## Supporting information

10.1192/j.eurpsy.2026.12234.sm001 Preiss et al. supplementary material Preiss et al. supplementary material

## Data Availability

The data supporting the findings of this study are not publicly available due to ethical and legal restrictions (GDPR) and the risk of re-identification in a single-center clinical registry. De-identified data may be made available from the corresponding author upon reasonable request and subject to approval by the data controller and the local ethics/data-protection governance. Analysis code is available from the corresponding author upon reasonable request.

## Supplementary material

The supplementary material for this article can be found at http://doi.org/10.1192/j.eurpsy.2026.12234.

## Use of artificial intelligence (AI) tools

In preparing this manuscript and supporting the development of the analysis pipeline, the authors used OpenAI ChatGPT (model GPT-5.2; accessed via the ChatGPT interface from September 2025 to February 2026). The tool was used for language and text editing, and to support programming tasks related to the statistical workflow. All AI-generated content was critically reviewed, edited, and approved by the authors; statistical analyses were executed by the authors, and all outputs were validated against the underlying scripts and source data. The tool was not used to generate or manipulate images. No identifiable patient information was entered into the tool.

## Long descriptions

### [Table 1] Long description

The table compares three groups: Induction population (n = 176), Induction completers (n = 156), and Induction dropouts (n = 20).

Key demographic and clinical variables include:

* Age: Mean of approximately 40.8 years across all groups.

* Sex: 54 percent female in the total population, 56.4 percent in completers, and 35 percent in dropouts.

* Diagnosis (I C D 10): Recurrent depression (F 33 dot x) is the primary diagnosis for over 93 percent of all groups.

* Depression Severity: Evenly split between Moderate (50.9 percent) and Severe (49.1 percent) in the total population.

* Clinical Scores: Mean M A D R S total score is 31.5. Mean P H Q 9 total score is 18.5. Median C G I S is 5.

* Treatment History: Median number of adequate A D trials is 5. Prior E C T was reported by 4.6 percent of the population.

* Comorbidities: Psychiatric comorbidity is present in 79.5 percent of the population. Anxiety disorder in the past year was present in 65.9 percent of the population, though notably lower in dropouts at 50 percent.

* Substance Use: 24.6 percent of the population had a substance use disorder in the past year, which was higher in the dropout group at 40 percent.

* Suicidality: Current suicidal ideation was present in 14.8 percent of the total population but 0 percent of the dropouts.

Navigate back to [Table 1].

### [Table 2] Long description

The table contains six columns: Class, Drug, Before baseline n (%), Before baseline mg/day mean (S D), After baseline n (%), and After baseline mg/day mean (S D).

* S S R I class: Escitalopram increased from 13 (7.4%) at 15.4 mg/day to 18 (10.2%) at 16.4 mg/day. Paroxetine increased from 4 (2.3%) to 6 (3.4%) at 33.3 mg/day. Sertraline increased from 12 (6.8%) at 141.7 mg/day to 15 (8.5%) at 150 mg/day.

* S N R I class: Duloxetine increased from 22 (12.5%) to 25 (14.2%). Milnacipran decreased from 15 (8.5%) to 13 (7.4%). Venlafaxine saw a significant increase from 55 (31.2%) at 234.5 mg/day to 88 (50%) at 258.1 mg/day.

* N D R I class: Bupropion changed from 41 (23.3%) at 307.3 mg/day to 38 (21.6%) at 323.7 mg/day.

* NaSSA class: Mirtazapine increased from 38 (21.6%) at 38.8 mg/day to 63 (35.8%) at 41.4 mg/day.

* S M S class: Vortioxetine changed from 14 (8%) at 27.1 mg/day to 15 (8.5%) at 15.7 mg/day.

* S A R I class: Trazodone decreased from 38 (21.6%) to 35 (19.9%).

* M A O I class: Moclobemide increased from 3 (1.7%) to 5 (2.8%) at 330 mg/day.

* M S class: Lamotrigine increased from 27 (15.3%) to 34 (19.3%). Lithium increased from 10 (5.7%) to 12 (6.8%).

* A A P class: Aripiprazole increased from 31 (17.6%) to 43 (24.4%). Quetiapine increased from 41 (23.3%) to 50 (28.4%).

* Other: Levothyroxine decreased from 11 (6.2%) to 10 (5.7%).

Navigate back to [Table 2].

### [Table 3] Long description

The table is divided into two primary outcome sections.

1. M A D R S total:

- M M R M adjusted mean at B L: Estimand 31.7, 95 percent C I 30.6 to 32.7.

- M M R M adjusted mean at P V: Estimand 30.3, 95 percent C I 29.2 to 31.3.

- M M R M adjusted mean at E O I: Estimand 17.7, 95 percent C I 16.2 to 19.3.

- Primary estimand (E O I minus P V): Estimand minus 12.5, 95 percent C I minus 14.1 to minus 11.0, P value less than 0.001, Hedges’ *g* 1.8.

2. P H Q minus 9 total:

- Piecewise mixed model early slope (V 1 to V 4): Estimand minus 0.6, 95 percent C I minus 0.8 to minus 0.5, P value less than 0.001.

- Piecewise mixed model late slope (V 4 to V 8): Estimand minus 0.8, 95 percent C I minus 0.9 to minus 0.6, P value less than 0.001.

- Late minus early slope difference: Estimand minus 0.1, 95 percent C I minus 0.4 to 0.1, P value 0.373.

- Secondary estimand (V 8 minus V 1): Estimand minus 4.9, 95 percent C I minus 5.7 to minus 4.2, P value less than 0.001, Hedges’ *g* 1.0.

Note: Negative values indicate symptom improvement. Abbreviations include M A D R S (Montgomery Asberg Depression Rating Scale) and P H Q minus 9 (Patient Health Questionnaire 9).

Navigate back to [Table 3].

### [Table 4] Long description

The table consists of seven columns: Outcome, Time point(s), Patient (n), Events (n), Estimate, 95% C I, and P value. It is divided into two main sections based on the Outcome.

1. Remission Outcome:

- P V: 166 patients, 1 event, 0.6% estimate, 95% C I of 0.0 to 3.3.

- E O I: 158 patients, 43 events, 27.2% estimate, 95% C I of 20.4 to 34.9.

- P V versus E O I (paired): 133 patients, P value less than 0.001.

2. Response Outcome:

- P V: 144 patients, 2 events, 1.4% estimate, 95% C I of 0.2 to 4.9.

- E O I: 136 patients, 78 events, 57.4% estimate, 95% C I of 48.6 to 65.8.

- P V versus E O I (paired): 133 patients, P value less than 0.001.

Footnotes indicate that percentages are calculated using the Patient (n) denominator. Response is defined as a M A D R S reduction of 50% or more from baseline, and Remission is defined as a M A D R S total score of 10 or less.

Navigate back to [Table 4].

### [Table 5] Long description

The table consists of five columns: Safety outcome, Patient-level incidence, 95% C I, Session-level incidence, and 95% C I.

* Any A E: Patient-level incidence is 34.7% (95% C I 27.7 to 42.2); Session-level incidence is 7.8% (95% C I 6.5 to 9.3).

* Blood-pressure-related events: Patient-level incidence is 10.2% (95% C I 6.2 to 15.7); Session-level incidence is 1.4% (95% C I 0.9 to 2.2).

* Dissociation: Patient-level incidence is 9.7% (95% C I 5.7 to 15.0); Session-level incidence is 2.0% (95% C I 1.3 to 2.9).

* Sedation: Patient-level incidence is 3.4% (95% C I 1.3 to 7.3); Session-level incidence is 0.8% (95% C I 0.4 to 1.4).

* Other A E: Patient-level incidence is 22.2% (95% C I 16.3 to 29.0); Session-level incidence is 5.5% (95% C I 4.4 to 6.9).

* Discontinuation due to A E: Patient-level incidence is 3.4% (95% C I 1.3 to 7.3); Session-level data is not applicable.

Note: Patient-level incidence denotes the proportion of patients with 1 or more event during induction. Session-level incidence denotes the proportion of induction administrations with an event recorded.

Navigate back to [Table 5].

### [Figure 1] Long description

The flowchart begins at the top with the Full Registry Cohort (n=229).

* A horizontal line to the right leads to Excluded (Did not meet inclusion criteria): (n=5).

* The main vertical line continues to the Pre-Induction Population (eligible): (n=224).

* A horizontal line to the right leads to Pre-Induction Dropouts: (n=48). Reasons include: 12 Not reached, 10 Inpatient induction, 9 Clinical improvement, 8 Lack of interest, 4 Hospitalization, 3 Esketamine initiated elsewhere, and 2 Pending treatment decision.

* The main vertical line continues to the Induction Population (greater than or equal to 1 esketamine treatment): (n=176).

* A horizontal line to the right leads to Induction-Phase Dropouts: (n=20). Reasons include: 7 Non-adherence, 6 Adverse events, 4 Lack of clinical response, 2 Somatic Illness, and 1 Hospitalization.

* The flowchart concludes at the bottom with Induction-Completers (greater than or equal to 8 esketamine treatments): (n=156).

Navigate back to [Figure 1].

### [Figure 2] Long description

The graph plots M A D R S total scores on the vertical Y axis, ranging from 0 to 50 in increments of 10. The horizontal X axis represents the Visit timeline with three discrete points: B L (Baseline), P V (Pre-treatment Visit), and E O I (End of Induction).

* The primary data is a thick blue line representing the model-based least-squares means. It starts at B L with a mean score of approximately 32, remains relatively stable at P V with a score of approximately 30, and then shows a sharp downward trend to E O I, ending at approximately 18. Each blue point includes vertical error bars representing 95 percent confidence intervals.

* Background data consists of numerous thin gray lines representing individual patient-level trajectories. These lines show high variability, with scores spread between 0 and 46 at B L.

* One notable outlier trajectory starts at 0 at B L, rises to approximately 14 at P V, and then drops back toward 0 at E O I.

* Most gray lines follow the general downward trend of the blue mean line between P V and E O I, though some individual scores remain high or increase.

Navigate back to [Figure 2].

### [Figure 3] Long description

The y-axis represents M A D R S item L S-mean scores from 0 to 6. The x-axis lists M A D R S items 1 through 10, grouped into three color-coded factor clusters. Each item has two overlapping bars: a lighter-shaded bar for the pre-induction visit (P V) and a darker-shaded bar for the end-of-induction (E O I). Error bars indicate 95 percent confidence intervals.

* Affective-Anhedonic Cluster (Blue): Includes items 1, 2, 7, and 8. P V scores are high, ranging from approximately 3.5 to 4.0. E O I scores show a significant reduction to approximately 2.0 for all items.

* Anxiety-Vegetative Cluster (Yellow): Includes items 3, 4, 5, and 6. P V scores vary from 1.4 (item 5) to 3.6 (item 6). E O I scores show consistent decreases, with item 5 dropping below 1.0.

* Hopelessness Cluster (Green): Includes items 9 and 10. Item 9 drops from a P V of 3.1 to an E O I of 1.7. Item 10 drops from a P V of 1.8 to an E O I of 0.9.

A legend on the right identifies the factors by color and the timepoints by shade and error bar style (grey for P V, black for E O I).

Navigate back to [Figure 3].

### [Figure 4] Long description

The horizontal X axis is labeled Visit and contains eight discrete points from V 1 to V 8. The vertical Y axis is labeled P H Q dash 9 total and ranges from 12 to 19 in increments of 1.

Data points and trends:

* The graph displays a downward linear trend starting at V 1 with a mean score of approximately 18.

* The line connects subsequent points at V 2 (approx. 17.4), V 3 (approx. 16.8), V 4 (approx. 16.2), V 5 (approx. 15.5), V 6 (approx. 14.7), V 7 (approx. 14.0), and ends at V 8 with a score of approximately 13.2.

* Each data point includes vertical error bars representing 95 percent confidence intervals.

* The line color transitions from a light blue shade at the beginning (V 1 to V 4) to a darker blue shade for the latter half (V 4 to V 8), indicating different treatment phases compared in the analysis.

Navigate back to [Figure 4].
