# Supplementary material for: Real-world effectiveness and safety of intranasal esketamine for treatment-resistant depression: Data from the enTRD registry
Source: Eur Psychiatry. 2026 Jun 23;69(1):e85. doi: 10.1192/j.eurpsy.2026.12234 (PMC13735771; doi:10.1192/j.eurpsy.2026.12234)
Supplement: Supplementary file 1 [file S0924933826122342sup001.docx]

Supplemental Material

Version 1.2,

04/09/2026

**Table of contents**

[Table 1: Baseline Characteristics by Population	4](#_Toc221580781)

[Table 2: Diagnoses stratified by Population	5](#_Toc221580782)

[Table 3: Antidepressant and Augmentation Regimen Change by Population	5](#_Toc221580783)

[Table 4: Medication by Drug Class and Name, Pre-Induction Population	5](#_Toc221580784)

[Table 5: Medication by Drug Class and Name, Induction Population	7](#_Toc221580785)

[Table 6: Average days in between Visits	9](#_Toc221580786)

[Table 7: Average Induction Treatment Esketamin Exposure	10](#_Toc221580787)

[Table 8: Primary Analysis Outcome	10](#_Toc221580788)

[Table 9: Sensitivity ANCOVA	10](#_Toc221580789)

[Table 10: Sensitivity MICE	10](#_Toc221580790)

[Table 11: Delta tipping point Analysis	10](#_Toc221580791)

[Table 12: LS-Means of MADRS factors	11](#_Toc221580792)

[Table 13: Contrasts between Affective-Anhedonic and both Affective-Anhedonic and Hopelessness factor	11](#_Toc221580793)

[Table 14: Association between Hopelessness and Overall MADRS Improvement during Induction	11](#_Toc221580794)

[Table 15: PHQ-9 change across induction session, early and late treatment phase	12](#_Toc221580795)

[Table 16: Response and Remission Rates at PV and EOI, Available Cases	12](#_Toc221580796)

[Table 17: Response and Remission Rates at PV and EOI, Non-Responder Imputation	12](#_Toc221580797)

[Table 18: McNemar analysis of Remission and Response, Pre-induction vs. Induction	13](#_Toc221580798)

[Table 19: Adverse Events during Induction Phase	13](#_Toc221580799)

[Table 20: LS-means of Visits of Self-Harm Ideation Item in MADRS and PHQ-9	13](#_Toc221580800)

Table 21: Response and Remission Rates at PV and EOI, Prior Neuromodulation…………………………………13
Table 22: Primary Effectiveness Outcome Excluding Patients with History of Hypomanic/Manic Episodes……...13
Table 23: Safety Summary Excluding Patients with History of Hypomanic/Manic Episodes……………………….14

[Figure 1: Adjusted MADRS item 10 Scores across Visits	1](#_Toc221581356)5

[Figure 2: Adjusted PHQ-9 item 9 Scores across Induction Sessions	1](#_Toc221581357)5

Figure 3: Study Timeline per Patient………………………………………………………………..…………………….16

Statistical Analysis Plan (SAP)…………………………………………………………………………….………………17

**Tables**:

Table 1: Baseline Characteristics by Population

| **Variable** | **Pre-Induction Population** | **Pre-Induction Dropouts** | **Induction Population** | **Induction Phase Dropouts** |
| --- | --- | --- | --- | --- |
| N patients | 224 | 48 | 176 | 20 |
| Age (years), mean (SD) | 41.9 (13.4) | 46.2 (15.1) | 40.8 (12.7) | 40.6 (12.3) |
| Sex, female, n (%) | 125 (55.8%) | 30 (62.5%) | 95 (54%) | 7 (35%) |
| Age at depression onset (years), mean (SD) | 23.8 (13.2) | 27.3 (16.9) | 22.7 (11.7) | 22.6 (8.6) |
| MADRS total, mean (SD) | 31.6 (6.3) | 31.8 (6.2) | 31.5 (6.4) | 30.2 (6.1) |
| PHQ-9 total, mean (SD) | 18.4 (4.7) | 18.3 (4.6) | 18.5 (4.7) | 17.5 (4.7) |
| CGI-S, median [IQR] | 5 [5; 6] | 5 [5; 6] | 5 [5; 6] | 5 [5; 6] |
| Adequate antidepressant trials (per-drug), median [IQR] | 5 [3; 6.2] | 4.5 [3.8; 6] | 5 [3; 7] | 5.5 [4; 6.2] |
| Adequate antidepressant classes, median [IQR] | 4 [3; 4] | 3 [2; 4] | 4 [3; 5] | 3.5 [3; 4.2] |
| Adequate augmentation trials (per-drug), median [IQR] | 1 [0; 2] | 1 [0; 2] | 1 [0; 2] | 1 [1; 2] |
| Current episode duration (months), median [IQR] | 12 [6; 23] | 12 [6; 19] | 12 [6; 24] | 17.5 [9; 29.5] |
| Prior ECT, n (%) | 10 (4.5%) | 2 (4.2%) | 8 (4.6%) | 2 (10.5%) |
| Prior VNS, n (%) | 1 (0.5%) | 0 (0%) | 1 (0.6%) | 0 (0%) |
| Prior esketamine, n (%) | 25 (11.5%) | 6 (12.8%) | 19 (11.1%) | 3 (16.7%) |
| Current suicidality, n (%) | 31 (13.8%) | 5 (10.4%) | 26 (14.8%) | 0 (0%) |
| History of severe suicidality, n (%) | 89 (40.3%) | 17 (37.8%) | 72 (40.9%) | 6 (30%) |
| Suicide attempts:  none  1  2  >2 | 156 (78.4%)  29 (14.6%)  11 (5.5%)  3 (1.5%) | 30 (78.9%)  3 (7.9%)  4 (10.5%)  1 (2.6%) | 126 (78.3%)  26 (16.1%)  7 (4.3%)  2 (1.2%) | 16 (88.9%)  1 (5.6%)  1 (5.6%)  0 (0%) |
| Inpatient admissions ≥2, n (%) | 94 (42.7%) | 22 (48.9%) | 72 (41.1%) | 7 (35%) |
| Outpatient-only care, n (%) | 201 (89.7%) | 38 (79.2%) | 163 (92.6%) | 19 (95%) |
| Psychiatric comorbidity, n (%) | 175 (78.5%) | 35 (74.5%) | 140 (79.5%) | 16 (80%) |
| Substance use disorder past year, n (%) | 55 (24.8%) | 12 (25.5%) | 43 (24.6%) | 8 (40%) |
| Anxiety disorder past year, n (%) | 146 (65.5%) | 30 (63.8%) | 116 (65.9%) | 10 (50%) |
| Somatic comorbidity, n (%) | 115 (51.6%) | 25 (53.2%) | 90 (51.1%) | 12 (60%) |
| Somatic risk: Hypertension, n (%) | 37 (16.7%) | 10 (21.7%) | 27 (15.3%) | 5 (25%) |
| Somatic risk: Hypothyroidism, n (%) | 32 (14.4%) | 9 (19.6%) | 23 (13.1%) | 2 (10%) |
| Somatic risk: Cerebrovascular history, n (%) | 7 (3.2%) | 3 (6.5%) | 4 (2.3%) | 1 (5%) |
| History of severe depressive episodes, n (%) | 170 (76.2%) | 33 (70.2%) | 137 (77.8%) | 17 (85%) |
| Lifetime number of depressive episodes, median [IQR] | 3 [2; 3.5] | 2.5 [1; 4] | 3 [2; 3.5] | 2 [2; 2.2] |
| Inter-episode remission, n (%) | 56 (31.8%) | 12 (41.4%) | 44 (29.9%) | 8 (47.1%) |
| Chronic MDD, n (%) | 156 (73.9%) | 28 (70%) | 128 (74.9%) | 16 (84.2%) |
| On SSRI or SNRI, n (%) | 186 (85.3%) | 43 (93.5%) | 143 (83.1%) | 17 (85%) |
| History of hypomanic/manic episode, n (%) | 26 (12.1%) | 9 (20.9%) | 17 (9.9%) | 3 (15%) |

*Legend: Data are in mean (SD), median [IQR] and n (%). MADRS, Montgomery-Asberg Depression Rating Scale; PHQ-9, Patient Health Questionnaire-9; CGI-S, Clinical Global Impression-Severity; AD, antidepressant; ECT, Electroconvulsive Therapy; VNS, Vagus Nerve Stimulation; EG, baseline appointment.*

Table 2: Diagnoses stratified by Population

| **Diagnosis** | **Pre-Induction** | **Pre-Induction Dropouts** | **Induction** | **Induction Dropouts** |
| --- | --- | --- | --- | --- |
| F31.3 | 3 (1.3%) | 1 (2.1%) | 2 (1.1%) | 1 (5%) |
| F31.4 | 8 (3.6%) | 4 (8.3%) | 4 (2.3%) | 0 (0%) |
| F31.6 | 1 (0.4%) | 0 (0%) | 1 (0.6%) | 0 (0%) |
| F32.1 | 2 (0.9%) | 0 (0%) | 2 (1.1%) | 0 (0%) |
| F32.2 | 2 (0.9%) | 0 (0%) | 2 (1.1%) | 0 (0%) |
| F33.0 | 1 (0.4%) | 1 (2.1%) | 0 (0%) | 0 (0%) |
| F33.1 | 96 (42.9%) | 12 (25%) | 84 (47.7%) | 9 (45%) |
| F33.2 | 109 (48.7%) | 29 (60.4%) | 80 (45.5%) | 10 (50%) |
| F33.4 | 1 (0.4%) | 0 (0%) | 1 (0.6%) | 0 (0%) |
| F25.1 | 1 (0.4%) | 1 (2.1%) | 0 (0%) | 0 (0%) |

Legend: Data are in n (%). Diagnoses assessed by clinician at Baseline.

Table 3: Antidepressant and Augmentation Regimen Change by Population

| **category** | **Pre-Induction Population, n (%)** | **Induction Population, n (%)** |
| --- | --- | --- |
| No change | 78 (34.8%) | 57 (32.4%) |
| AD dose only | 18 (8.0%) | 17 (9.7%) |
| AUG dose only | 1 (0.4%) | 1 (0.6%) |
| Both AD and AUG dose only | 4 (1.8%) | 3 (1.7%) |
| AD start only | 46 (20.5%) | 37 (21.0%) |
| AD stop only | 5 (2.2%) | 4 (2.3%) |
| AD switch | 22 (9.8%) | 16 (9.1%) |
| AUG start only | 10 (4.5%) | 8 (4.5%) |
| AUG stop only | 3 (1.3%) | 3 (1.7%) |
| Both AD and AUG regimen change | 37 (16.5%) | 30 (17.0%) |

*Legend: Data are in n (%). Table reflects regimen changes made at Baseline visit. Data are in n (%). AD antidepressant, AUG Augmentation.*

Table 4: Medication by Drug Class and Name, Pre-Induction Population

| **Class** | **Drug** | **Before BL**  **n (%);**  **dose mean (SD)** | **After BL**  **n (%);**  **dose mean (SD)** | **Ever (max)**  **n (%);**  **dose mean (SD)** | **Adequate duration n (% among ever)** |
| --- | --- | --- | --- | --- | --- |
| Antipsychotic | Clozapine | 1 (0.4%);  – | 1 (0.4%);  – | 1 (0.4%);  – | 1 (100%) |
| Atypical antipsychotic | Amisulpride | 2 (0.9%);  – | 1 (0.4%);  – | 11 (4.9%);  88.6 (105.7) | 11 (100%) |
| Atypical antipsychotic | Aripiprazole | 35 (15.6%);  8.9 (6.5) | 49 (21.9%);  7.7 (4.9) | 101 (45.1%);  7.4 (4.9) | 89 (88.1%) |
| Atypical antipsychotic | Cariprazine | 3 (1.3%);  – | 3 (1.3%);  – | 6 (2.7%);  2.2 (0.8) | 5 (83.3%) |
| Atypical antipsychotic | Olanzapine | 6 (2.7%);  7.5 (5) | 6 (2.7%);  6.7 (4.9) | 31 (13.8%);  6.5 (3.8) | 28 (90.3%) |
| Atypical antipsychotic | Quetiapine | 53 (23.7%);  194.3 (154.5) | 63 (28.1%);  179.2 (152.6) | 128 (57.1%);  155.3 (155.5) | 122 (95.3%) |
| Atypical antipsychotic | Risperidone | 6 (2.7%);  1.8 (1.3) | 3 (1.3%);  – | 22 (9.8%);  1.8 (1.9) | 20 (90.9%) |
| Benzo-diazepine | Alprazolam | 6 (2.7%);  0.7 (0.2) | 5 (2.2%);  0.8 (0.4) | 14 (6.2%);  0.9 (0.4) | 10 (71.4%) |
| Benzo-diazepine | Clonazepam | 6 (2.7%);  0.9 (0.6) | 14 (6.2%);  0.8 (0.3) | 17 (7.6%);  1 (0.7) | 14 (82.4%) |
| Benzo-diazepine | Lorazepam | 5 (2.2%);  1.6 (1.5) | 5 (2.2%);  2.3 (1.6) | 10 (4.5%);  3.2 (2.9) | 7 (70%) |
| Benzo-diazepine | Oxazepam | 2 (0.9%);  – | 2 (0.9%);  – | 5 (2.2%);  60.5 (62) | 4 (80%) |
| MAOI | Moclobemide | 3 (1.3%);  – | 5 (2.2%);  330 (67.1) | 18 (8%);  358.3 (163.8) | 14 (77.8%) |
| MAOI | Tranylcypromine | 0 (0%);  – | 0 (0%);  – | 6 (2.7%);  30 (16.7) | 4 (66.7%) |
| Mood stabilizer | Lamotrigine | 32 (14.3%);  223.4 (128.4) | 39 (17.4%);  203.8 (125.1) | 73 (32.6%);  161.6 (135.7) | 69 (94.5%) |
| Mood stabilizer | Lithium | 13 (5.8%);  900 (343.7) | 16 (7.1%);  759.4 (375.4) | 36 (16.1%);  675 (331.6) | 33 (91.7%) |
| Mood stabilizer | Topiramate | 4 (1.8%);  – | 5 (2.2%);  120 (185.7) | 8 (3.6%);  88.1 (147.3) | 7 (87.5%) |
| Mood stabilizer | Valproate | 0 (0%);  – | 1 (0.4%);  – | 10 (4.5%);  890 (242.4) | 9 (90%) |
| NDRI | Bupropion | 51 (22.8%);  305.9 (108) | 47 (21%);  316 (95) | 149 (66.5%);  309.1 (95.9) | 143 (96%) |
| NRI | Reboxetine | 1 (0.4%);  – | 1 (0.4%);  – | 11 (4.9%);  4.2 (1.4) | 10 (90.9%) |
| NaSSA | Mianserin | 1 (0.4%);  – | 2 (0.9%);  – | 15 (6.7%);  44 (21.9) | 13 (86.7%) |
| NaSSA | Mirtazapine | 47 (21%);  38.7 (16.4) | 75 (33.5%);  41 (17.3) | 157 (70.1%);  36.4 (19.3) | 144 (91.7%) |
| Other | Agomelatin | 1 (0.4%);  – | 1 (0.4%);  – | 7 (3.1%);  35.7 (13.4) | 4 (57.1%) |
| Other | Flupentixol/  Melitracen | 6 (2.7%);  5.2 (7.8) | 2 (0.9%);  – | 16 (7.1%);  1.9 (1.2) | 16 (100%) |
| Other | Hydroxyzine | 1 (0.4%);  – | 3 (1.3%);  – | 2 (0.9%);  – | 2 (100%) |
| Other | John's Wort | 1 (0.4%);  – | 1 (0.4%);  – | 9 (4%);  611.1 (231.5) | 7 (77.8%) |
| Other | Levothyroxine | 12 (5.4%);  55.2 (49.2) | 13 (5.8%);  59.6 (38.9) | 25 (11.2%);  63.9 (43.2) | 23 (92%) |
| Other | Methylphenidate | 12 (5.4%);  50.8 (69.6) | 14 (6.2%);  36.3 (24.5) | 15 (6.7%);  49.3 (62.2) | 14 (93.3%) |
| Other | Naltrexon | 1 (0.4%);  – | 1 (0.4%);  – | 1 (0.4%);  – | 1 (100%) |
| Other | Pramipexole | 2 (0.9%);  – | 1 (0.4%);  – | 1 (0.4%);  – | 1 (100%) |
| Other | Pregabalin | 47 (21%);  313.8 (223.8) | 53 (23.7%);  297.2 (210.7) | 68 (30.4%);  321.3 (244.1) | 62 (91.2%) |
| Other | Propranolol | 2 (0.9%);  – | 2 (0.9%);  – | 2 (0.9%);  – | 2 (100%) |
| Other | Prothipendyl-hydrochlorid | 18 (8%);  80 (33.6) | 15 (6.7%);  88 (34.5) | 25 (11.2%);  89.6 (38.8) | 20 (80%) |
| Other | Triiodothyronine | 0 (0%);  – | 0 (0%);  – | 0 (0%);  – | 0 (NA) |
| Other | Zolpidem | 14 (6.2%);  9.5 (2) | 13 (5.8%);  9.4 (2.1) | 22 (9.8%);  9.4 (1.9) | 21 (95.5%) |
| SARI | Trazodone | 48 (21.4%);  120.8 (58.4) | 45 (20.1%);  129.4 (74.7) | 140 (62.5%);  207 (132.9) | 132 (94.3%) |
| SMS | Vortioxetin | 16 (7.1%);  24.7 (33.8) | 23 (10.3%);  13.3 (6) | 48 (21.4%);  13.4 (5.7) | 36 (75%) |
| SNRI | Duloxetine | 26 (11.6%);  90 (36) | 30 (13.4%);  93 (34.7) | 114 (50.9%);  75 (31.5) | 106 (93%) |
| SNRI | Milnacipran | 17 (7.6%);  105.9 (34.8) | 14 (6.2%);  101.8 (47.5) | 59 (26.3%);  103 (38) | 52 (88.1%) |
| SNRI | Venlafaxine | 63 (28.1%);  240.5 (100.3) | 103 (46%);  258.4 (75.5) | 180 (80.4%);  226.7 (80.9) | 159 (88.3%) |
| SSRI | Citalopram | 6 (2.7%);  25 (8.4) | 4 (1.8%);  – | 34 (15.2%);  24 (9.5) | 33 (97.1%) |
| SSRI | Escitalopram | 21 (9.4%);  17.6 (11.5) | 25 (11.2%);  17.8 (10.7) | 149 (66.5%);  14.1 (6.9) | 144 (96.6%) |
| SSRI | Fluoxetine | 9 (4%);  57.8 (18.6) | 10 (4.5%);  50 (23.6) | 62 (27.7%);  31.3 (19.1) | 58 (93.5%) |
| SSRI | Fluvoxamine | 0 (0%);  – | 0 (0%);  – | 6 (2.7%);  59.2 (47.2) | 3 (50%) |
| SSRI | Paroxetine | 4 (1.8%);  – | 6 (2.7%);  33.3 (10.3) | 33 (14.7%);  28.9 (13.4) | 30 (90.9%) |
| SSRI | Sertraline | 18 (8%);  138.9 (88.4) | 21 (9.4%);  145.2 (80.5) | 125 (55.8%);  105.2 (61.2) | 119 (95.2%) |
| TCA | Amitriptyline | 3 (1.3%);  – | 3 (1.3%);  – | 36 (16.1%);  116.5 (57.2) | 33 (91.7%) |
| TCA | Clomipramine | 7 (3.1%);  101.8 (60.6) | 8 (3.6%);  101.6 (52.8) | 25 (11.2%);  127 (51.4) | 24 (96%) |
| TCA | Maprotiline | 1 (0.4%);  – | 0 (0%);  – | 6 (2.7%);  139.6 (72.6) | 4 (66.7%) |
| TCA | Opipramol | 1 (0.4%);  – | 1 (0.4%);  – | 5 (2.2%);  140 (22.4) | 3 (60%) |
| TCA | Tianeptin | 4 (1.8%);  – | 11 (4.9%);  40.9 (11.3) | 44 (19.6%);  44 (15.3) | 33 (75%) |

*Legend: Concomitant psychopharmacotherapy in the pre-induction population before and after baseline. Medications are grouped by drug class and individual agent. For each agent, use is summarised as n (%) and mean daily dose (SD) for the period before baseline (Before BL) and after baseline during the pre-induction interval (After BL). “Ever (max)” indicates any exposure at any time before BL, with the maximum documented daily dose per patient summarised as n (%) and mean (SD). Adequate duration is reported as n (%) among patients ever exposed to the agent, according to prespecified adequacy criteria. Mean daily doses are reported only when at least five patients contributed dose data for the respective agent and time window; otherwise, only n (%) is shown. MAOI, monoamine oxidase inhibitor; NDRI, norepinephrine–dopamine reuptake inhibitor; NRI, norepinephrine reuptake inhibitor; NaSSA, noradrenergic and specific serotonergic antidepressant; SARI, serotonin antagonist and reuptake inhibitor; SMS, second messenger system modulator; SNRI, serotonin–norepinephrine reuptake inhibitor; SSRI, selective serotonin reuptake inhibitor; TCA, tricyclic antidepressant. “Atypical antipsychotic” refers to second-generation antipsychotics, and “Benzodiazepine” refers to benzodiazepines.*

Table 5: Medication by Drug Class and Name, Induction Population

| **Class** | **Drug** | **Before BL**  **n (%);**  **dose mean (SD)** | **After BL**  **n (%);**  **dose mean (SD)** | **Ever (max)**  **n (%);**  **dose mean (SD)** | **Adequate duration n (% among ever)** |
| --- | --- | --- | --- | --- | --- |
| Antipsychotic | Clozapine | 0 (0%);  – | 0 (0%);  – | 0 (0%);  – | 0 (NA) |
| Atypical antipsychotic | Amisulpride | 2 (1.1%);  – | 1 (0.6%);  – | 9 (5.1%);  97.2 (116.2) | 9 (100%) |
| Atypical antipsychotic | Aripiprazole | 31 (17.6%);  9.1 (6.8) | 43 (24.4%);  7.9 (5.1) | 78 (44.3%);  7.8 (5.4) | 69 (88.5%) |
| Atypical antipsychotic | Cariprazine | 3 (1.7%);  – | 3 (1.7%);  – | 6 (3.4%);  2.2 (0.8) | 5 (83.3%) |
| Atypical antipsychotic | Olanzapine | 4 (2.3%);  – | 3 (1.7%);  – | 27 (15.3%);  6.8 (4) | 25 (92.6%) |
| Atypical antipsychotic | Quetiapine | 41 (23.3%);  199.1 (161.4) | 50 (28.4%);  180.5 (153.2) | 99 (56.2%);  152.7 (159.8) | 93 (93.9%) |
| Atypical antipsychotic | Risperidone | 4 (2.3%);  – | 2 (1.1%);  – | 16 (9.1%);  1.6 (1.4) | 15 (93.8%) |
| Benzo-diazepine | Alprazolam | 4 (2.3%);  – | 3 (1.7%);  – | 8 (4.5%);  0.8 (0.4) | 5 (62.5%) |
| Benzo-diazepine | Clonazepam | 5 (2.8%);  0.9 (0.6) | 10 (5.7%);  0.8 (0.4) | 11 (6.2%);  1.2 (0.8) | 10 (90.9%) |
| Benzo-diazepine | Lorazepam | 5 (2.8%);  1.6 (1.5) | 5 (2.8%);  2.3 (1.6) | 7 (4%);  1.9 (1.1) | 6 (85.7%) |
| Benzo-diazepine | Oxazepam | 2 (1.1%);  – | 1 (0.6%);  – | 4 (2.3%);  – | 3 (75%) |
| MAOI | Moclobemide | 3 (1.7%);  – | 5 (2.8%);  330 (67.1) | 16 (9.1%);  365.6 (172.9) | 12 (75%) |
| MAOI | Tranylcypromine | 0 (0%);  – | 0 (0%);  – | 6 (3.4%);  30 (16.7) | 4 (66.7%) |
| Mood stabilizer | Lamotrigine | 27 (15.3%);  224.1 (132.7) | 34 (19.3%);  202.9 (127) | 58 (33%);  171.6 (136.5) | 55 (94.8%) |
| Mood stabilizer | Lithium | 10 (5.7%);  922.5 (326.1) | 12 (6.8%);  768.8 (377.2) | 28 (15.9%);  683 (338.1) | 25 (89.3%) |
| Mood stabilizer | Topiramate | 4 (2.3%);  – | 5 (2.8%);  120 (185.7) | 8 (4.5%);  88.1 (147.3) | 7 (87.5%) |
| Mood stabilizer | Valproate | 0 (0%);  – | 1 (0.6%);  – | 7 (4%);  842.9 (282) | 6 (85.7%) |
| NDRI | Bupropion | 41 (23.3%);  307.3 (105.8) | 38 (21.6%);  323.7 (89.1) | 117 (66.5%); 310.3 (95.9) | 112 (95.7%) |
| NRI | Reboxetine | 1 (0.6%);  – | 1 (0.6%);  – | 10 (5.7%);  3.8 (0.6) | 9 (90%) |
| NaSSA | Mianserin | 1 (0.6%);  – | 2 (1.1%);  – | 12 (6.8%);  45 (23.5) | 10 (83.3%) |
| NaSSA | Mirtazapine | 38 (21.6%);  38.8 (15.4) | 63 (35.8%);  41.4 (18) | 126 (71.6%);  37.5 (19.6) | 115 (91.3%) |
| Other | Agomelatin | 1 (0.6%);  – | 1 (0.6%);  – | 7 (4%);  35.7 (13.4) | 4 (57.1%) |
| Other | Flupentixol/  Melitracen | 4 (2.3%);  – | 2 (1.1%);  – | 10 (5.7%);  2.1 (1.4) | 10 (100%) |
| Other | Hydroxyzine | 0 (0%);  – | 1 (0.6%);  – | 1 (0.6%);  – | 1 (100%) |
| Other | John's Wort | 1 (0.6%);  – | 1 (0.6%);  – | 8 (4.5%);  612.5 (247.5) | 6 (75%) |
| Other | Levothyroxine | 11 (6.2%);  51.1 (49.4) | 10 (5.7%);  52.5 (41.6) | 18 (10.2%);  57.6 (39.5) | 16 (88.9%) |
| Other | Methylphenidate | 11 (6.2%);  54.5 (71.7) | 13 (7.4%);  35.2 (25.1) | 14 (8%);  49.3 (64.5) | 13 (92.9%) |
| Other | Naltrexon | 1 (0.6%);  – | 1 (0.6%);  – | 1 (0.6%);  – | 1 (100%) |
| Other | Pramipexole | 2 (1.1%);  – | 1 (0.6%);  – | 1 (0.6%);  – | 1 (100%) |
| Other | Pregabalin | 36 (20.5%);  290.3 (178.5) | 38 (21.6%);  295.4 (172.3) | 47 (26.7%);  344.7 (229.7) | 43 (91.5%) |
| Other | Propranolol | 1 (0.6%);  – | 1 (0.6%);  – | 1 (0.6%);  – | 1 (100%) |
| Other | Prothipendyl-hydrochlorid | 16 (9.1%);  82.5 (34.2) | 14 (8%);  91.4 (33) | 22 (12.5%);  94.5 (38.1) | 18 (81.8%) |
| Other | Triiodothyronine | 0 (0%);  – | 0 (0%);  – | 0 (0%);  – | 0 (NA) |
| Other | Zolpidem | 11 (6.2%);  9.3 (2.3) | 10 (5.7%);  9.2 (2.4) | 13 (7.4%);  9.4 (2.1) | 12 (92.3%) |
| SARI | Trazodone | 38 (21.6%);  125.7 (61.6) | 35 (19.9%);  138.6 (79.4) | 110 (62.5%); 211.8 (127.1) | 104 (94.5%) |
| SMS | Vortioxetin | 14 (8%);  27.1 (35.6) | 15 (8.5%);  15.7 (5.9) | 38 (21.6%);  14.3 (5.7) | 31 (81.6%) |
| SNRI | Duloxetine | 22 (12.5%);  96.8 (34.6) | 25 (14.2%);  98.4 (33) | 93 (52.8%);  76.1 (31.8) | 86 (92.5%) |
| SNRI | Milnacipran | 15 (8.5%);  110 (33.8) | 13 (7.4%);  101.9 (49.4) | 48 (27.3%);  103.1 (38.8) | 42 (87.5%) |
| SNRI | Venlafaxine | 55 (31.2%);  234.5 (100.3) | 88 (50%);  258.1 (75.3) | 145 (82.4%); 229.7 (81.7) | 127 (87.6%) |
| SSRI | Citalopram | 4 (2.3%);  – | 2 (1.1%);  – | 29 (16.5%);  24 (9.8) | 28 (96.6%) |
| SSRI | Escitalopram | 13 (7.4%);  15.4 (5.2) | 18 (10.2%);  16.4 (6.4) | 115 (65.3%);  14.1 (5.9) | 110 (95.7%) |
| SSRI | Fluoxetine | 5 (2.8%);  52 (22.8) | 6 (3.4%);  40 (25.3) | 52 (29.5%);  30 (18) | 48 (92.3%) |
| SSRI | Fluvoxamine | 0 (0%);  – | 0 (0%);  – | 5 (2.8%);  59 (52.7) | 2 (40%) |
| SSRI | Paroxetine | 4 (2.3%);  – | 6 (3.4%);  33.3 (10.3) | 28 (15.9%);  27.9 (11) | 25 (89.3%) |
| SSRI | Sertraline | 12 (6.8%);  141.7 (101.9) | 15 (8.5%);  150 (88.6) | 101 (57.4%);  104 (62.3) | 96 (95%) |
| TCA | Amitriptyline | 1 (0.6%);  – | 1 (0.6%);  – | 30 (17%);  122.7 (56.5) | 28 (93.3%) |
| TCA | Clomipramine | 4 (2.3%);  – | 5 (2.8%);  95 (51.2) | 19 (10.8%);  125.7 (54.1) | 18 (94.7%) |
| TCA | Maprotiline | 0 (0%);  – | 0 (0%);  – | 5 (2.8%);  162.5 (51.5) | 4 (80%) |
| TCA | Opipramol | 1 (0.6%);  – | 1 (0.6%);  – | 4 (2.3%);  – | 2 (50%) |
| TCA | Tianeptin | 4 (2.3%);  – | 8 (4.5%);  42.2 (13.3) | 36 (20.5%);  44.4 (15.9) | 29 (80.6%) |

*Legend: Concomitant psychopharmacotherapy in the induction population before and after baseline. Medications are grouped by drug class and individual agent. For each agent, use is summarised as n (%) and mean daily dose (SD) for the period before baseline (Before BL) and after baseline during the pre-induction interval (After BL). “Ever (max)” indicates any exposure at any time before BL, with the maximum documented daily dose per patient summarised as n (%) and mean (SD). Adequate duration is reported as n (%) among patients ever exposed to the agent, according to prespecified adequacy criteria. Mean daily doses are reported only when at least five patients contributed dose data for the respective agent and time window; otherwise, only n (%) is shown. MAOI, monoamine oxidase inhibitor; NDRI, norepinephrine–dopamine reuptake inhibitor; NRI, norepinephrine reuptake inhibitor; NaSSA, noradrenergic and specific serotonergic antidepressant; SARI, serotonin antagonist and reuptake inhibitor; SMS, second messenger system modulator; SNRI, serotonin–norepinephrine reuptake inhibitor; SSRI, selective serotonin reuptake inhibitor; TCA, tricyclic antidepressant. “Atypical antipsychotic” refers to second-generation antipsychotics.*

Table 6: Average days in between Visits

| **Interval (days)** | **Median [IQR]** | **N** |
| --- | --- | --- |
| BL to EOI | 125.5 [72; 207] | 164 |
| BL to PV | 96 [43; 183] | 153 |
| PV to EOI | 30 [28.8; 37] | 152 |

*Legend: Intervals between study visits (days). For each interval (BL–PV, PV–EOI, and BL–EOI), the table reports the number of patients with available date information (N) and the median time in days with interquartile range (IQR). BL denotes the baseline visit, PV the pre-treatment visit immediately preceding the first intranasal esketamine administration, and EOI the end-of-induction visit following completion or discontinuation of the induction phase.*

Table 7: Average Induction Treatment Esketamine Exposure

| **Variable** | **Induction Population** | **Induction Completer** | **Induction Phase Dropouts** |
| --- | --- | --- | --- |
| Induction treatments, median [IQR] | 8 [4;8] | 8 [8;8] | 4 [2.5; 6] |
| Induction dose total, mean (SD) | 461.7 (257.1) | 569.3 (141.2) | 254.9 (175.6) |

*Legend: Average treatment frequency and dosage by population. Induction treatments are reported as the median number of administrations with interquartile range (IQR). Total induction dose is reported as the mean cumulative dose across induction administrations with standard deviation (SD).*

Table 8: Primary Analysis Outcome

| **Outcome** | **Timepoint** | **Contrast** | **Estimate** | **CI 95%** | **P Value** |
| --- | --- | --- | --- | --- | --- |
| MADRS total score | BL | - | 31.67 | 30.64 to 32.69 | - |
| MADRS total score | PV | - | 30.25 | 29.20 to 31.30 | - |
| MADRS total score | EOI | - | 17.73 | 16.16 to 19.29 | - |
| Primary effect | - | EOI − PV | -12.53 | -14.05 to -11.01 | p<0.0001 |
| Effect size | - | Hedges g (EOI−PV) | -1.8 | - | - |

*Legend: Primary outcome (MADRS) least-squares means and treatment effect from the primary MMRM. The table reports adjusted mean MADRS total scores at baseline (BL), pre-treatment visit (PV), and end of induction (EOI), the primary contrast (EOI − PV) with 95% confidence interval and P Value, and the corresponding effect size (Hedges g).*

Table 9: Sensitivity ANCOVA

| **Estimand** | **Estimate** | **SE** | **df** | **t** | **Lower CL** | **Upper CL** | **N** | **P Value** |
| --- | --- | --- | --- | --- | --- | --- | --- | --- |
| Adjusted mean change PV-EOI | -12.3 | 0.750 | 138 | -16.3 | -13.7 | -10.8 | 143 | p<0.0001 |

*Legend: Sensitivity analysis using ANCOVA for change in MADRS from pre-treatment visit (PV) to end of induction (EOI). The table reports the adjusted mean PV–EOI change estimated from an ANCOVA model of MADRS at EOI with MADRS at PV and prespecified covariates. Standard error (SE), degrees of freedom (df), test statistic (t), 95% confidence limits (CL), analysis sample size (N).*

Table 10: Sensitivity MICE

| **Estimand** | **Estimate** | **Lower CL** | **Upper CL** | **SE** | **m** | **maxit** | **P Value** |
| --- | --- | --- | --- | --- | --- | --- | --- |
| PV-EOI | -12.6 | -14.2 | -11.1 | 0.784 | 20 | 50 | p<0.0001 |

Legend: Sensitivity analysis using multiple imputation by chained equations (MICE) for the PV–EOI change in MADRS. The table reports the pooled estimate and standard error (SE) with 95% confidence limits (CL) and P Value after imputing missing MADRS values under a missing-at-random assumption and refitting the prespecified model in each imputed dataset. The number of imputations (m) and iterations (maxit) are shown.

Table 11: Delta tipping point Analysis

| **delta** | **Estimate** | **Lower CL** | **Upper CL** | **SE** | **m** | **P Value** |
| --- | --- | --- | --- | --- | --- | --- |
| 0 | -12.63 | -14.17 | -11.09 | 0.78 | 20 | p<0.0001 |
| 0.5 | -12.58 | -14.12 | -11.04 | 0.78 | 20 | p<0.0001 |
| 1 | -12.54 | -14.07 | -11.00 | 0.78 | 20 | p<0.0001 |
| 1.5 | -12.49 | -14.03 | -10.95 | 0.78 | 20 | p<0.0001 |
| 2 | -12.44 | -13.98 | -10.91 | 0.78 | 20 | p<0.0001 |
| 2.5 | -12.40 | -13.93 | -10.86 | 0.78 | 20 | p<0.0001 |
| 3 | -12.35 | -13.89 | -10.81 | 0.78 | 20 | p<0.0001 |
| 3.5 | -12.30 | -13.84 | -10.77 | 0.79 | 20 | p<0.0001 |
| 4 | -12.26 | -13.80 | -10.72 | 0.79 | 20 | p<0.0001 |
| 4.5 | -12.21 | -13.76 | -10.67 | 0.79 | 20 | p<0.0001 |
| 5 | -12.17 | -13.71 | -10.62 | 0.79 | 20 | p<0.0001 |
| 5.5 | -12.12 | -13.67 | -10.57 | 0.79 | 20 | p<0.0001 |
| 6 | -12.07 | -13.62 | -10.52 | 0.79 | 20 | p<0.0001 |
| 6.5 | -12.03 | -13.58 | -10.48 | 0.79 | 20 | p<0.0001 |
| 7 | -11.98 | -13.54 | -10.43 | 0.79 | 20 | p<0.0001 |
| 7.5 | -11.94 | -13.50 | -10.38 | 0.80 | 20 | p<0.0001 |
| 8 | -11.89 | -13.45 | -10.33 | 0.80 | 20 | p<0.0001 |
| 8.5 | -11.84 | -13.41 | -10.28 | 0.80 | 20 | p<0.0001 |
| 9 | -11.80 | -13.37 | -10.23 | 0.80 | 20 | p<0.0001 |
| 9.5 | -11.75 | -13.33 | -10.18 | 0.80 | 20 | p<0.0001 |
| 10 | -11.71 | -13.29 | -10.12 | 0.81 | 20 | p<0.0001 |

*Legend: Delta tipping point means incrementally increasing imputed outcome values by 0.5 MADRS points and refitting the model. This accounts as sanity check for data missing at random assumptions. Standard error (SE)* *with 95% confidence limits (CL), imputation iterations (m), and P Value are reported.*

Table 12: LS-Means of MADRS factors

| **factor** | **Visit** | **Estimate** | **Lower CL** | **Upper CL** | **SE** | **df** |
| --- | --- | --- | --- | --- | --- | --- |
| Affective-Anhedonic | BL | 3.68 | 3.54 | 3.82 | 0.07 | 1,124.00 |
| Affective-Anhedonic | PV | 3.63 | 3.48 | 3.78 | 0.08 | 1,124.00 |
| Affective-Anhedonic | EOI | 2.04 | 1.83 | 2.24 | 0.10 | 1,124.00 |
| Anxiety-Vegetative | BL | 2.89 | 2.75 | 3.04 | 0.07 | 1,124.00 |
| Anxiety-Vegetative | PV | 2.66 | 2.51 | 2.82 | 0.08 | 1,124.00 |
| Anxiety-Vegetative | EOI | 1.63 | 1.43 | 1.83 | 0.10 | 1,124.00 |
| Hopelessness | BL | 2.81 | 2.67 | 2.95 | 0.07 | 1,124.00 |
| Hopelessness | PV | 2.44 | 2.29 | 2.60 | 0.08 | 1,124.00 |
| Hopelessness | EOI | 1.27 | 1.07 | 1.47 | 0.10 | 1,124.00 |

*Legend: Least-squares means of MADRS factor scores across visits. The table reports model-based adjusted mean scores for the affective–anhedonic, anxiety–vegetative, and hopelessness factors at baseline (BL), pre-treatment visit (PV), and end of induction (EOI), with standard errors (SE), degrees of freedom (df), and 95% confidence limits (CL). Factor scores represent the mean of items within each cluster (range 0–6).*

Table 13: Contrasts between Affective-Anhedonic and both Affective-Anhedonic and Hopelessness factor

| **Contrast** | **Estimate** | **Lower CL** | **Upper CL** | **SE** | **df** | **T ratio** | **P Value** |
| --- | --- | --- | --- | --- | --- | --- | --- |
| Δ_affective –  Δ_anxiety | -0.56 | -0.72 | -0.39 | 0.08 | 189.00 | -6.65 | p<0.0001 |
| Δ_affective - Δ_hopelessness | -0.51 | -0.67 | -0.34 | 0.08 | 189.00 | -6.06 | p<0.0001 |

*Legend: Between-factor contrasts in change from PV to EOI. The table reports differences in the PV–EOI change (Δ) between the affective–anhedonic factor and the anxiety–vegetative factor, and between the affective–anhedonic factor and the hopelessness factor. Negative estimates indicate a larger reduction (greater improvement) in the affective–anhedonic factor relative to the comparison factor. Estimates are presented with standard errors (SE), degrees of freedom (df), t ratios, P Values, and 95% confidence limits (CL).*

Table 14: Association between Hopelessness and Overall MADRS Improvement during Induction

| **term** | **OR per 1SD** | **Lower CL** | **Upper CL** | **P Value** | **N** | **events** |
| --- | --- | --- | --- | --- | --- | --- |
| Hopelessness ~ MADRS improvement | 1.54 | 0.93 | 2.54 | p=0.0907 | 127 | 63 |

*Legend: Exploratory logistic regression examining whether change in the hopelessness factor (per 1 standard deviation) was associated with overall MADRS improvement. Results are presented as odds ratios (OR) per 1 SD with 95% confidence limits (CL) and P Value. N denotes the analysis sample size and events the number of patients meeting the predefined improvement criterion.*

Table 15: PHQ-9 change across induction session, early and late treatment phase

| **segment** | **estimate** | **Lower CL** | **Upper CL** | **SE** | **P Value** |
| --- | --- | --- | --- | --- | --- |
| Early slope  (V1–V4) | -0.64 | -0.82 | -0.46 | 0.09 | p<0.0001 |
| Late slope  (V5–V8) | -0.75 | -0.90 | -0.61 | 0.08 | p<0.0001 |
| Total Difference  (V8-V1) | -4.93 | -4.20 | -5.67 | 0.37 | p<0.0001 |
| Difference  (late − early) | -0.12 | -0.37 | 0.14 | 0.13 | p=0.3729 |

*Legend: Piecewise mixed-model slopes for PHQ-9 change across induction sessions. The table reports the adjusted per-session change (slope) in PHQ-9 total score during the early segment V1-V4 and late segment V5-V8, and the between-segment difference (late − early), with standard errors (SE), degrees of freedom (df), P Values, and 95% confidence limits (CL).*

Table 16: Response and Remission Rates at PV and EOI, Available Cases

| **comparison** | **n** | **category** | **events** | **%** | **Lower CL** | **Upper CL** |
| --- | --- | --- | --- | --- | --- | --- |
| BL to PV | 166 | Remission | 1 | 0.6 | 0.0 | 3.3 |
|  | 144 | Response≥50% | 2 | 1.4 | 0.2 | 4.9 |
|  |  | Response≥30% | 15 | 10.4 | 5.9 | 16.6 |
|  |  | No response | 127 | 88.2 | 81.8 | 93 |
| BL to EOI | 158 | Remission | 43 | 27.2 | 20.4 | 34.9 |
|  | 136 | Response≥50% | 78 | 57.4 | 48.6 | 65.8 |
|  |  | Response≥30% | 16 | 11.8 | 6.9 | 18.4 |
|  |  | No response | 42 | 30.9 | 23.2 | 39.4 |
| PV to EOI | 158 | Remission | 43 | 27.2 | 20.4 | 34.9 |
|  | 153 | Response≥50% | 73 | 47.7 | 39.6 | 55.9 |
|  |  | Response≥30% | 27 | 17.6 | 12 | 24.6 |
|  |  | No response | 53 | 34.6 | 27.1 | 42.7 |

*Legend: Response and remission were derived from MADRS total scores and are presented for changes from baseline (BL) to pre-treatment visit (PV), BL to end of induction (EOI), and PV to EOI. Response was defined as a ≥50% reduction in MADRS relative to the respective starting time point, and non-response as <30% reduction. Remission was defined separately as MADRS ≤10 at the respective end time point and is therefore not mutually exclusive with response categories. Percentages are calculated using available cases for each comparison, with exact 95% confidence limits (CL).*

Table 17: Response and Remission Rates at PV and EOI, Non-Responder Imputation

| **comparison** | **n** | **category** | **events** | **%** | **Lower CL** | **Upper CL** |
| --- | --- | --- | --- | --- | --- | --- |
| BL to PV | 176 | Remission | 1 | 0.6 | 0.0 | 3.1 |
|  |  | Response≥50% | 2 | 1.1 | 0.1 | 4 |
|  |  | Response≥30% | 15 | 8.5 | 4.8 | 13.7 |
|  |  | No response | 159 | 90.3 | 85 | 94.3 |
| BL to EOI |  | Remission | 43 | 24.4 | 18.3 | 31.5 |
|  |  | Response≥50% | 78 | 44.3 | 36.8 | 52 |
|  |  | Response≥30% | 16 | 9.1 | 5.3 | 14.3 |
|  |  | No response | 82 | 46.6 | 39.1 | 54.2 |
| PV to EOI |  | Remission | 43 | 24.4 | 18.3 | 31.5 |
|  |  | Response≥50% | 73 | 41.5 | 34.1 | 49.1 |
|  |  | Response≥30% | 27 | 15.3 | 10.4 | 21.5 |
|  |  | No response | 76 | 43.2 | 35.8 | 50.8 |

*Legend: Response and remission were derived from MADRS total scores and are presented for changes from baseline (BL) to pre-treatment visit (PV), BL to end of induction (EOI), and PV to EOI. Response was defined as a ≥50% reduction in MADRS relative to the respective starting time point, and non-response as <30% reduction. Remission was defined separately as MADRS ≤10 at the respective end time point and is therefore not mutually exclusive with response categories. Percentages are calculated using available cases, all missing cases and dropouts are counted as Non Responder, with exact 95% confidence limits (CL).*

Table 18: McNemar analysis of Remission and Response, Pre-Induction vs. Induction

| **Category** | **Endpoint** | **N paired** | **McNemar X²** | **P Value** |
| --- | --- | --- | --- | --- |
| Remission | PV vs. EOI | 133 | 34.0 | p<0.0001 |
| Response >50% | PV vs. EOI | 133 | 74.0 | p<0.0001 |

*Legend: McNemar’s χ² tests were used to compare paired remission status (MADRS ≤10) and response ≥50% between the pre-treatment visit (PV) and end of induction (EOI) among patients with outcome data at both time points (N paired). P Values are shown for the null hypothesis of no change in the paired proportions.*

Table 19: Adverse Events during Induction Phase

| **Endpoint** | **level** | **n** | **%** | **Lower CL** | **Upper CL** |
| --- | --- | --- | --- | --- | --- |
| Any AE | per patient | 61 | 34.66 | 27.66 | 42.19 |
| Dissociation |  | 17 | 9.66 | 5.73 | 15.01 |
| Sedation |  | 6 | 3.41 | 1.26 | 7.27 |
| RR-related |  | 18 | 10.23 | 6.17 | 15.68 |
| Other |  | 39 | 22.16 | 16.26 | 29.02 |
| Any AE | overall (per session) | 110 | 7.81 | 6.46 | 9.34 |
| Dissociation |  | 28 | 1.99 | 1.33 | 2.86 |
| Sedation |  | 11 | 0.78 | 0.39 | 1.39 |
| RR-related |  | 20 | 1.42 | 0.87 | 2.19 |
| Other |  | 78 | 5.54 | 4.40 | 6.87 |

*Legend: The table summarises adverse events (AEs) recorded during induction, presented both per patient (proportion of patients with ≥1 event in the category) and per session (proportion of administrations with an event). Estimates are reported as n and percentage with exact 95% confidence limits (CL). RR-related denotes blood pressure–related events, and “Other” includes adverse events not classified as dissociation, sedation, or RR-related like severe hallucinations, nausea, or vomiting.*

Table 20: LS-means of Visits of Self-Harm Ideation Item in MADRS and PHQ-9

| **outcome** | **Visit** | **Estimate** | **Lower CL** | **Upper CL** | **SE** | **df** | **P Value** |
| --- | --- | --- | --- | --- | --- | --- | --- |
| MADRS, Item10 | PV | 0.90 | 0.68 | 1.12 | 0.11 | 251 | - |
|  | EOI | 1.77 | 1.55 | 1.99 | 0.11 | 251 |  |
|  | EOI-PV | -0.87 | -1.10 | -0.63 | 0.12 | 251 | p<0.0001 |
| PHQ-9, Item 9 | V01 | 1.20 | 1.04 | 1.35 | 0.08 | 1,208.00 | - |
|  | V02 | 1.24 | 1.08 | 1.40 | 0.08 | 1,208.00 |  |
|  | V03 | 1.10 | 0.94 | 1.26 | 0.08 | 1,208.00 |  |
|  | V04 | 1.01 | 0.85 | 1.17 | 0.08 | 1,208.00 |  |
|  | V05 | 1.00 | 0.85 | 1.16 | 0.08 | 1,208.00 |  |
|  | V06 | 0.90 | 0.75 | 1.05 | 0.07 | 1,208.00 |  |
|  | V07 | 0.87 | 0.72 | 1.01 | 0.07 | 1,208.00 |  |
|  | V08 | 0.81 | 0.67 | 0.96 | 0.07 | 1,208.00 |  |
|  | V08-V01 | -0.38 | -0.52 | -0.25 | 0.07 | 1,208.00 | p<0.0001 |

*Legend: The table presents least-squares means (adjusted estimates) at the specified time points and the corresponding contrasts from the prespecified mixed models. MADRS item 10 (suicidal thoughts; range 0–6) is reported at the pre-treatment visit (PV) and end of induction (EOI) with the primary contrast (EOI − PV). PHQ-9 item 9 (thoughts of self-harm or being better off dead; range 0–3) is summarised across induction sessions (V01–V08) in the session-level model. Estimates are shown with standard errors (SE), degrees of freedom (df), and 95% confidence limits (CL).*

Table 21: Response and Remission Rates at PV and EOI, Prior Neuromodulation

| **Endpoint** | **Group** | **N** | **Events** | **%** | **Lower CL** | **Upper CL** | **P Value** |
| --- | --- | --- | --- | --- | --- | --- | --- |
| Remission at EOI | No prior neuromodulation | 138 | 38 | 27.5 | 20.3 | 35.8 | p=1 |
| Remission at EOI | Prior neuromodulation | 5 | 1 | 20 | 0.5 | 71.6 |  |
| Response at EOI | No prior neuromodulation | 133 | 61 | 45.9 | 37.2 | 54.7 | p=0.663 |
| Response at EOI | Prior neuromodulation | 5 | 3 | 60 | 14.7 | 94.7 |  |

*Legend: Response and remission were derived from MADRS total scores and are presented for changes from pre-treatment visit (PV) to end of induction (EOI). Response was defined as a ≥50% reduction in MADRS relative to the respective starting time point. Remission was defined separately as MADRS ≤10 at EOI and is therefore not mutually exclusive with response. Percentages are calculated using available cases, with exact 95% confidence limits (CL).*

Table 22: Primary Effectiveness Outcome Excluding Patients with History of Hypomanic/Manic Episodes

| **Outcome** | **Contrast** | **Estimate** | **CI 95%** | **P Value** |
| --- | --- | --- | --- | --- |
| Primary effect | EOI−PV | −12.3 | −13.9 to −10.7 | p<0.0001 |
| Effect size, Hedges’ g | EOI−PV | −1.74 | – | – |

*Primary outcome (MADRS) least-squares treatment effect from the MMRM, excluding patients with a documented history of hypomanic or manic episodes (n = 17 excluded; analysis sample n = 159). The model specification is identical to the primary analysis (Table 8). The table reports the primary contrast (EOI − PV) with 95% confidence interval and P Value, and the corresponding effect size (Hedges’ g).*

Table 23: Safety Summary Excluding Patients with History of Hypomanic/Manic Episodes

| **Endpoint** | **Level** | **n** | **%** | **Lower CL** | **Upper CL** |
| --- | --- | --- | --- | --- | --- |
| Any AE | per patient (N=159) | 57 | 35.8 | 28.4 | 43.8 |
| Dissociation |  | 16 | 10.1 | 5.9 | 15.8 |
| Sedation |  | 4 | 2.5 | 0.7 | 6.3 |
| RR-related |  | 16 | 10.1 | 5.9 | 15.8 |
| Other |  | 38 | 23.9 | 17.5 | 31.3 |
| Any AE | per session (N=1272) | 103 | 8.1 | 6.7 | 9.7 |
| Dissociation |  | 26 | 2.0 | 1.3 | 3.0 |
| Sedation |  | 8 | 0.6 | 0.3 | 1.2 |
| RR-related |  | 18 | 1.4 | 0.8 | 2.2 |
| Other |  | 74 | 5.8 | 4.6 | 7.2 |

*Adverse events (AEs) during induction, excluding patients with a documented history of hypomanic or manic episodes (n = 17 excluded; per-patient denominator N = 159, per-session denominator N = 1272). Presentation follows the format of the full-sample safety table (Table 19). Estimates are reported as n and percentage with exact 95% confidence limits (CL). RR-related denotes blood-pressure–related events; “Other” includes events not classified as dissociation, sedation, or RR-related (e.g., nausea, vomiting, hallucinations).*

**Figures**

Figure 1: Adjusted MADRS item 10 Scores across Visits

*Legend: Lines show model-based least-squares means for MADRS item 10 (suicidal thoughts; range 0–6) at pre-treatment visit (PV) and end of induction (EOI). Error bars indicate 95% confidence intervals. The PV estimate reflects symptom severity immediately prior to the first intranasal esketamine administration, and the EOI estimate reflects symptom severity at the end of the induction phase.*

Figure 2: Adjusted PHQ-9 item 9 Scores across Induction Treatment Sessions

Legend: *Lines show model-based least-squares means for PHQ-9 item 9 (thoughts of self-harm or being better off dead; range 0–3) across induction sessions V1–V8. Error bars indicate 95% confidence intervals. Session-level assessment was collected as part of routine care at each administration.*

Figure 3: Study Timeline per Patient

**enTRD Registry — Study Timeline per Patient: Pre-Induction and Induction Phase**

*Monocentric registry study | TRD Outpatient Clinic, Department of Psychiatry and Psychotherapy, Medical University of Vienna*

BL

PV

V1

V2

V3

V4

V5

V6

V7

V8

**EOI**

**AD optimization**

Exclude pseudo-resistance

**Assessments**

MADRS (BL, PV, EOI) | YMRS (BL) | CGI-S (all visits)

**Self-Report**

PHQ-9, VAS-Mood (BL, V1–V8, EOI) | GAD-7 (BL, EOI)

**Safety / Tolerability**

BP monitoring, clinical assessment of side effects (all treatment visits) | FIBSER (BL, EOI)

**Diagnostic / Staging**

MINI, MDQ (BL) | MSM, ATHF-SF, ATRQ (BL)

**Quality of Life**

EQ-5D-5L, WSAS (BL, EOI)

**Lab / Somatic**

Drug screening, routine labs, vitals (BL)

**Patient Flow**

Referred (n=229) → Eligible (n=224) → Initiated ESK (n=176) → Induction Completers (n=156) | Dropouts: Pre-induction n=48, Induction n=20

*AD, antidepressant; BL, baseline; BP, blood pressure; EOI, end of induction; ESK, esketamine; PV, pre-treatment visit; V, treatment visit. ATHF-SF, Antidepressant Treatment History Form, Short Form; ATRQ, Antidepressant Treatment Response Questionnaire; CGI-S, Clinical Global Impression–Severity; EQ-5D-5L, EuroQol 5-Dimension 5-Level; FIBSER, Frequency, Intensity, and Burden of Side Effects Rating; GAD-7, Generalized Anxiety Disorder 7-item; MADRS, Montgomery–Åsberg Depression Rating Scale; MDQ, Mood Disorder Questionnaire; MINI, Mini-International Neuropsychiatric Interview; MSM, Maudsley Staging Method; PHQ-9, Patient Health Questionnaire-9; VAS, Visual Analogue Scale; WSAS, Work and Social Adjustment Scale; YMRS, Young Mania Rating Scale.*

**Intranasal esketamine (Spravato®, up to 84 mg)**

+ oral AD (SSRI/SNRI)

**Pre-Induction**

(~1–4 Months)

**Induction Phase**

(4 Weeks, 2×/Week)

**Treatment**

**Clinician-Rated**

**Statistischer Analyseplan (SAP)**

**Titel: Wiener Patientenregister und Beobachtungsstudie für therapieresistente Depression (enTRD)**

**Title: Vienna Patient Registry and observational study of treatment resistant depression (enTRD)**

Version 1.1, Versionsdatum 04.01.2026

# 1. Allgemeines

## 1.1. Metadaten

| **Autoren d. SAP** | Assoc.Prof. Priv.Doz. Dr. Lukas Pezawas Dr. Med. Univ. Maximilian Preiß |
| --- | --- |
| **Art des Dokuments** | Statistischer Analyseplan (SAP) |
| **Seitenzahl** | 60 |

## 1.2. Standard Definitionen und Abkürzungen

| **Abbreviation** | **Meaning** |
| --- | --- |
| AE | Adverse Event |
| CI | Confidence Interval / Konfidenzintervall |
| E | Effektstärke |
| EG | Eingangsuntersuchung (Baseline) |
| enTRD | Esketamin‑TRD‑Registerstudie |
| FAS | Full Analysis Set |
| Hedges g | Effektstärke nach Hedges |
| ID | Teilnehmerkennung |
| ITT | Intention‑to‑Treat |
| LS‑Mean | Least‑Squares‑Mittelwert / Modelladjustierter Mittelwert |
| MADRS | Montgomery‑Åsberg Depression Rating Scale |
| MI | Multiple Imputation |
| MMRM | Mixed‑Model for Repeated Measures |
| NU | Nachuntersuchung |
| OR | Odds Ratio |
| PHQ‑9 | Patient Health Questionnaire‑9 |
| PP | Per‑Protocol |
| RR‑AE | Rapid‑Response‑Adverse‑Event (Schwellenwert im SAP definiert) |
| SAE | Serious Adverse Event |
| SAP | Statistischer Analyseplan |
| SD | Standard Deviation / Standardabweichung |
| VU | Voruntersuchung |

# 2. Zusammenfassung

## 2.1. Abstrakt

Der Statistische Analyseplan (SAP) der enTRD‑Registerstudie definiert die in der aktuellen Version vorgesehenen Auswertungen zur Veränderung depressiver Symptomatik, zu klinischen Verlaufsparametern sowie zu Sicherheitsereignissen während der Esketamin‑Induktion. Der SAP legt die Analysepopulationen, die statistischen Modelle und die Methoden zur Behandlung fehlender Daten fest und folgt einem modularen Aufbau.

Der primäre Auswertungsfokus liegt auf der Veränderung des MADRS-Gesamtscores zwischen Voruntersuchung (VU) und Nachuntersuchung (NU; Ende der Induktionsphase), geschätzt mittels eines Mixed-Models für wiederholte Messungen (MMRM) mit den Messzeitpunkten Eingangsuntersuchung (EG), VU und NU. Zur Prüfung der Robustheit werden ein baseline-adjustiertes Modell mit MADRS-Gesamtscore bei EG als Kovariate sowie Auswertungen nach Multipler Imputation (MICE) und pattern-mixture-Modellen prä-spezifiziert. Sekundäre Analysen umfassen Veränderungen der drei MADRS‑Faktoren, deren Vergleich untereinander sowie die prognostische Bedeutung des Hoffnungslosigkeitsfaktors für ein Therapieansprechen. Der Verlauf der selbstberichteten Depressivität (PHQ‑9) über die Induktionsphase wird mittels eines linearen gemischten Modell untersucht. Zusätzlich werden Response‑ und Remissionsraten, Sicherheitsparameter und suizidalitätsrelevante Ereignisse deskriptiv berichtet. Fehlende Werte werden im Rahmen von mixed models unter der Annahme Missing at Random (MAR) behandelt; ergänzend sind Sensitivitätsanalysen vorgesehen.

Der SAP wird versioniert geführt. Analysen, die über den in Version 1.1 definierten Umfang hinausgehen, werden ausschließlich durch ein formelles Amendement ergänzt.

## 2.2. Abstract

The Statistical Analysis Plan (SAP) of the enTRD registry study outlines the analyses included in the current version, specifying the statistical methods, analysis populations, modelling approaches, and handling of missing data. The SAP follows a modular structure, in which additional analyses may be incorporated through formally approved amendments issued prior to the start of any new analysis modules.

The primary analytical focus is the change in clinician‑rated depressive symptoms, operationalized as the MADRS total score difference between the pre‑treatment assessment (VU) and post‑induction assessment (NU; end of Induction phase), analyzed using a mixed-model for repeated measures (MMRM) including MADRS total scores at baseline (EG), pre-treatment assessment (VU), and post-induction assessment (NU). Robustness will be evaluated using a baseline-adjusted model with MADRS at EG as covariate, as well as multiple imputation (MICE) and pattern-mixture models. Secondary analyses include changes in the three predefined MADRS factor scores, comparisons between factor‑specific improvements, and assessment of baseline hopelessness as a predictor of treatment response. Patient‑reported depressive symptoms (PHQ‑9) during the induction phase are analyzed via a piecewise linear mixed‑effects model. Response and remission rates, safety outcomes, and suicidality‑related events are summarized descriptively. Missing data are handled through mixed‑effects modelling under a Missing at Random (MAR) assumption, with additional sensitivity analyses defined.

SAP Version 1.1 reflects all analyses pre‑specified prior to data evaluation.

# 3. Inhaltsverzeichnis

[1. Allgemeines	1](#_Toc1827436203)

[1.1. Metadaten	2](#_Toc1417783322)

[1.2. Standard Definitionen und Abkürzungen	2](#_Toc1246499502)

[2. Zusammenfassung	2](#_Toc2052144398)

[2.1. Abstrakt	3](#_Toc40913977)

[2.2. Abstract	3](#_Toc2083810404)

[3. Inhaltsverzeichnis	3](#_Toc653705593)

[4. Einleitung und Zweck	4](#_Toc869802285)

[5. Analysepopulationen	5](#_Toc301055828)

[5.1. Deskriptives Analyseset	5](#_Toc1923506920)

[5.2. Induktions-Analysepopulation	5](#_Toc1552861106)

[6. Primärer statistischer Endpunkt	5](#_Toc1119604218)

[6.1. Definition	5](#_Toc764141154)

[6.2. Analytische Endpunkte	6](#_Toc1947509916)

[6.3. Statistisches Modell	6](#_Toc1451108514)

[7. Sekundäre Endpunkte A – MADRS‑Faktoren	6](#_Toc1862833855)

[7.1. Sekundärer Endpunkt A1	6](#_Toc427911380)

[7.2. Sekundärer Endpunkt A2	7](#_Toc51716519)

[7.3. Sekundärer Endpunkt A3	8](#_Toc833841382)

[8. Sekundäre Endpunkte B - Longitudinales Selbstrating (PHQ-9)	8](#_Toc207696301)

[8.1. Sekundärer Endpunkt B1	8](#_Toc122534069)

[8.2. Sekundärer Endpunkt B2	8](#_Toc1006706635)

[9. Sekundäre Endpunkte C– Response und Remission	8](#_Toc1064190530)

[10. Sekundäre Endpunkte D - Sicherheit und Suizidalität	8](#_Toc852598686)

[11. Umgang mit fehlenden Daten	8](#_Toc845739603)

[12. Signifikanzniveau	8](#_Toc1682428012)

[13. Software	8](#_Toc1198345802)

[14. Modularität und Amendments	8](#_Toc1617363844)

[15. Literaturverzeichnis	8](#_Toc661766778)

# 4. Einleitung und Zweck

Dieser Statistische Analyseplan (SAP) legt die in der aktuellen Version geplanten statistischen Auswertungen der enTRD-Registerstudie fest. Er definiert die verwendeten Analysepopulationen, statistischen Methoden, Modelle, Kontraste sowie den Umgang mit fehlenden Daten.

Der SAP ist **modular aufgebaut**: **SAP Version 1.1** enthält ausschließlich diejenigen Analysen, die vor Beginn der jeweiligen Auswertungsphase verbindlich festgelegt wurden.

# 5. Analysepopulationen

## 5.1. Deskriptives Analyseset

Alle Patient:innen mit mindestens einem initialen TRD-Abklärungsanamnese und dokumentierter Indikation für intranasales Esketamin (d. h. in den Behandlungsweg aufgenommen, unabhängig davon, ob die Esketamin-Behandlung tatsächlich begonnen wurde).

## 5.2. Induktions-Analysepopulation

Alle Patient:innen, die mindestens eine intranasale Esketamin-Behandlung erhalten haben; diese Population dient als Hauptkollektiv für die Analysen zur Wirksamkeit (MADRS, PHQ-9) und Sicherheit in der Induktionsphase, wobei alle vorhandenen Post-Baseline-Daten genutzt werden.

# 6. Primärer statistischer Endpunkt

## 6.1. Definition

Primärer Endpunkt ist die Veränderung der ärztlich bewerteten depressiven Symptomatik (MADRS Gesamtscore) in der Induktions-Analysepopulation zwischen:

- **Voruntersuchung (VU)** und
- **Nachuntersuchung (NU)**,

Die Veränderung VU → NU wird als modelladjustierte Differenz der LS-Means zwischen VU und NU aus einem Mixed-Model für wiederholte Messungen (MMRM) mit den Zeitpunkten EG, VU und NU definiert unter Adjustierung für Alter und Geschlecht. Der primäre Effekt ist der Kontrast NU vs. VU.

## 6.2. Analytische Endpunkte

- LSMean MADRS sum score zu EG, VU und NU (95 %-Konfidenzintervall)^1^, adjustiert für Alter und Geschlecht
- LSMean Change VU → NU (adjustiert für Alter und Geschlecht)
- Effektstärke (Hedges g)
- Sensitivitätsanalysen:
  - **Baseline-adjustiertes Modell:** ANCOVA mit Veränderung zu Nachuntersuchung (NU) als Endpunkt und VU, Alter, Gender, und PHQ9 Wert bei VU als Selbstwahrnehmung: *Delta_MADRSVU_NU_ ∼MADRS_VU_ +Alter+Geschlecht+PHQ9_VU_*
  - **Multiple Imputation (MICE):** Gemeinsame Imputation der MADRS-Werte zu EG, VU und NU sowie relevanter Kovariaten unter MAR-Annahme; Wiederholung des primären MMRM in jeder imputierten Stichprobe und Pooling der NU-vs-VU-Kontraste nach Rubin.

**Pattern-mixture-Modelle:** Durchführung von Delta-Adjustments / Tipping-Point-Analysen zur Prüfung der Robustheit gegenüber MNAR-Mechanismen.

## 6.3. Statistisches Modell

Die Primäranalyse des MADRS-Gesamtscores erfolgt mittels Mixed-Model for Repeated Measures (MMRM) in der Induktions-Analysepopulation.

**Modellspezifikation:**
 abhängige Variable: MADRS_total
 Zeitfaktor: Visit (kategorial; Stufen EG, VU, NU)
 Kovariaten: Alter, Geschlecht

Formal:

*MADRS_total∼Visit_EG_+Visit_VU_+Visit_NU_+Alter+Geschlecht+ε*

Dabei wird Visit ohne globalen Intercept parametrisiert (entsprechend separaten Mittelwerten je Visit).

Für die Kovarianzstruktur der wiederholten Messungen wird eine unstrukturierte Kovarianzmatrix mit besuchsabhängigen Varianzen verwendet.

**Primärer Effekt** ist der Kontrast NU vs. VU aus den modellbasierten LS-Means adjustiert für Alter und Geschlecht.

# 7. Sekundäre Endpunkte A – MADRS‑Faktoren

Die Analysen basieren auf einer vorab festgelegten dreifaktoriellen Struktur des MADRS^2^:

- Affektiv–Anhedonisch (Fragen: 1,2,7,8)
- Angst-Vegetativ (Fragen: 3,4,5,6)
- Hoffnungslosigkeit (Fragen: 9,10)

Für jeden Faktor wird der Score als *Mean-per-item* berechnet (Skala 0–6), um die Vergleichbarkeit zwischen Faktoren mit unterschiedlicher Itemanzahl sicherzustellen:
Affektiv–Anhedonisch = (Items 1,2,7,8)/4;

Angst–Vegetativ = (Items 3,4,5,6)/4;

Hoffnungslosigkeit = (Items 9,10)/2.
Summenscores können zusätzlich deskriptiv berichtet werden; inferenzstatistische Analysen basieren auf Mean-per-item.

## 7.1. Sekundärer Endpunkt A1

Definition:

Sekundärer Endpunkt A1 ist die Veränderung der ärztlich bewerteten depressiven Symptomatik auf Ebene der drei MADRS-Faktoren (Affektiv-Anhedonisch, Ängstlich-Vegetativ, Hoffnungslosigkeit) in der Induktions-Analysepopulation zwischen Voruntersuchung (VU) und Nachuntersuchung (NU). Zusätzlich werden die Verläufe der drei Faktoren über die Messzeitpunkte EG, VU und NU beschrieben.

Analytische Endpunkte für jeden der drei MADRS-Faktoren:

- LSMean Faktor-Score zu EG, VU und NU (95 %-Konfidenzintervall) adjustiert für Alter und Geschlecht
- LSMean Change VU → NU (modellbasiert, *adjustiert für Alter und Geschlecht*)
- Effektstärke (Hedges g) für die Veränderung VU → NU
- Globaler Test des Zeiteffekts (VU vs. NU) über alle Faktoren sowie faktor­spezifische Tests, ob die Veränderung VU → NU ungleich 0 ist (sekundär/explorativ, ohne primäre Alpha-Kontrolle)

Statistisches Modell:

MMRM mit gemeinsamem Modell für alle drei Faktoren:

*MADRS_faktor_i_∼Zeit*Faktor+Alter+Geschlecht+ ε*

- *MADRS_faktor_i_*: MADRS-Faktor-Score (Affektiv-Anhedonisch, Ängstlich-Vegetativ, Hoffnungslosigkeit) zu EG, VU bzw. NU
- Zeit: kategorial (EG, VU, NU)
- Faktor: kategorial (3 Stufen: Affective–Anhedonic, Anxiety–Vegetative, Hopelessness)

Für die Kovarianzstruktur der wiederholten Messungen wird – analog zum Primärmodell – eine unstrukturierte Kovarianzmatrix mit ggf. besuchsabhängigen Varianzen verwendet. Alle verfügbaren Faktor-Messwerte gehen unter der Annahme MAR in das Modell ein; Patient:innen können auch dann beitragen, wenn einzelne Messzeitpunkte fehlen.

Der Haupteffekt von time und die einfachen Effekte von time innerhalb jedes Faktors liefern die modellbasierten LS-Means und LS-Mean-Changes (insbesondere VU → NU) je Faktor.

## 7.2. Sekundärer Endpunkt A2

Definition:

Sekundärer Endpunkt A2 ist der Unterschied in der Veränderung der drei MADRS-Faktoren (Affektiv-Anhedonisch, Ängstlich-Vegetativ, Hoffnungslosigkeit) zwischen Voruntersuchung (VU) und Nachuntersuchung (NU), wobei insbesondere geprüft wird, ob die Verbesserung im Affektiv-Anhedonischen Faktor größer ist als in den beiden anderen Faktoren. Zur Prüfung, ob die Verbesserung im Affektiv–Anhedonischen Faktor über das hinausgeht, was durch höhere Ausgangswerte erklärbar ist, wird A2 als baseline-adjustierter Vergleich der Faktor-Changes modelliert.

Analytische Endpunkte:

Differenz der modelladjustierten (für Alter und Geschlecht) LSMean-Changes VU → NU zwischen

- Affective–Anhedonic und Anxiety–Vegetative (Δ_affective − Δ_anxiety),
- Affective–Anhedonic und Hopelessness (Δ_affective − Δ_hopelessness),
   jeweils mit 95 %-Konfidenzintervall und p-Wert.

Statistisches Modell:

Für jede Patient:in *i* und jeden Faktor *f* wird Δ_{i,f} = (NU_{i,f} − VU_{i,f}) berechnet.
 Modell:
 Δ_{i,f} = μ + Faktor_f + β·VU_{i,f} + γ1·Alter_i + γ2·Geschlecht_i + u_i + ε_{i,f}

mit u_i als patientenspezifischem Random Intercept (Korrelation der 3 Faktor-Changes innerhalb einer Person).

Aus dem Zeit×Faktor-Interaktionsterm werden die baseline-adjustierten LSMean-Changes VU → NU je Faktor abgeleitet und anschließend als lineare Kontraste die Differenzen Δ_affective − Δ_anxiety und Δ_affective − Δ_hopelessness mit 95 %-Konfidenzintervallen und p-Werten geschätzt.

## 7.3. Sekundärer Endpunkt A3

Definition:

Sekundärer Endpunkt A3 ist der Zusammenhang zwischen der Höhe des MADRS-Hopelessness-Faktors vor Behandlung (VU) und der Wahrscheinlichkeit eines Ansprechens am Ende der Induktionsphase.
Ansprechen (Response) wird definiert als ≥50 % Reduktion des MADRS-Gesamtscores von der Voruntersuchung (VU) bis zur Nachuntersuchung (NU) in der Induktions-Analysepopulation.

Analytische Endpunkte:

- Odds Ratio (OR) für Nicht-Ansprechen an NU pro 1-Standardabweichung (1 SD) höherem Hopelessness-Faktor bei VU, mit 95 %-Konfidenzintervall und p-Wert, adjustiert für die Ausgangsschwere (MADRS Gesamtscore bei VU) sowie Alter und Geschlecht.

Statistisches Modell:

Logistisches Regressionsmodell in der Induktions-Analysepopulation:

*Non_Response_NU_ ~ Hopelessness_VUz_ + MADRS_VU_ + Alter + Geschlecht*

- Non_Response_NU: binärer Endpunkt (1 = <50 % Reduktion MADRS_total VU → NU, 0 = Ansprechen)
- Hopelessness_VU_z: z-standardisierter MADRS-Hopelessness-Faktor bei VU (Mittelwert 0, SD 1)
- MADRS_VU: MADRS-Gesamtscore bei VU (Kontrolle der Ausgangsschwere)

Die logistische Regression verwendet den Logit-Link, Schätzung mittels Maximum-Likelihood. Der Haupteffekt von Hopelessness_VU_z liefert die OR pro 1 SD höherem Hopelessness-Ausgangswert für Non-Response bei NU.

# 8. Sekundäre Endpunkte B - Longitudinales Selbstrating (PHQ-9)

## 8.1. Sekundärer Endpunkt B1

Definition:

Sekundärer Endpunkt B1 ist der Verlauf der selbstbewerteten depressiven Symptomatik (PHQ-9-Gesamtscore) über die Induktionsphase (trt01–trt08) in der Induktions-Analysepopulation. Ziel ist, Ausmaß und Form der Symptomveränderung bis zur Mitte (trt04) und zum Ende (trt08) der Induktion zu quantifizieren. Die PHQ-9-Bewertung bei der Eingangs­untersuchung (EG) wird deskriptiv berichtet. **^1,3^**

Analytische Endpunkte:

- LSMean des PHQ-9 Gesamtscores für jede Induktionssitzung (trt01–trt08) mit 95 %-Konfidenzintervall, adjustiert für Alter und Geschlecht.
- LSMean-Change trt01 → trt04 mit 95 %-Konfidenzintervall.
- LSMean-Change trt01 → trt08 mit 95 %-Konfidenzintervall.
- Standardisierte Effektstärken (Hedges g) für die Veränderungen trt01 → trt04 und trt01 → trt08, und trt01→ trt08 jeweils mit 95 %-Konfidenzintervall.

Statistisches Modell:

MMRM mit partierter Zeitabbildung (früh/spät):

*PHQ9_total ~ t_early + t_late + Alter + Geschlecht + ε*

- PHQ9_total: PHQ-9 Gesamtscore zu den Induktionssitzungen trt01–trt08
- time: numerischer Sitzungsindex (1 = trt01, …, 8 = trt08)
- t_early = pmin(time, 4): Zeitkomponente für die frühe Phase (trt01–trt04)
- t_late = pmax(0, time − 4): zusätzliche Zeitkomponente für die späte Phase (trt05–trt08)

Aus diesem Modell werden die LSMeans pro Sitzung sowie die LSMean-Changes trt01 → trt04 und trt01 → trt08 und die entsprechenden Effektstärken abgeleitet. (Dasselbe Modell wird in B2 zur formalen Prüfung der frühen vs. späten Steigung verwendet.)

## 8.2. Sekundärer Endpunkt B2

Definition:

Sekundärer Endpunkt B2 ist der Vergleich des Ausmaßes der Symptomverbesserung in der frühen (trt01–trt04) und späten Induktionsphase (trt04–trt08), gemessen am Verlauf des PHQ-9 Gesamtscores. Es soll geprüft werden, ob die mittlere Abnahmerate (Slope) in der frühen Phase sich statistisch von derjenigen in der späten Phase unterscheidet (H₀: frühe und späte Steigung sind gleich).

Analytische Endpunkte:

- Geschätzte frühe Steigung (Slope) des PHQ-9 pro Sitzung von trt01 bis trt04 mit 95 %-Konfidenzintervall.
- Geschätzte späte Steigung (Slope) des PHQ-9 pro Sitzung von trt04 bis trt08 mit 95 %-Konfidenzintervall.
- Differenz der Steigungen (Slope_spät − Slope_früh) mit 95 %-Konfidenzintervall und p-Wert (Test der Nullhypothese: keine Differenz der Steigungen)

Statistisches Modell:

Für B2 wird dasselbe stückweise Mixed Model for Repeated Measures verwendet wie für B1.

In diesem Modell entspricht der Koeffizient von t_early der frühen Steigung; die späte Steigung ergibt sich als Summe der Koeffizienten von t_early und t_late. Der Koeffizient von t_late selbst repräsentiert die Differenz der Steigungen (Slope_spät − Slope_früh); ein Wald-Test auf t_late = 0 liefert den p-Wert für B2.

# 9. Sekundäre Endpunkte C– Response und Remission

Definition:

Sekundärer Endpunkt C sind MADRS-basierte **Response- und Remissionsraten** in der Induktions-Analysepopulation zu Voruntersuchung (VU) und Nachuntersuchung (NU), jeweils **relativ zur Einganguntersuchung (EG)** als Baseline.

- **Teil-Response:** ≥30 % Reduktion des MADRS-Gesamtscores von EG zu VU bzw. NU.
- **Response**: ≥50 % Reduktion des MADRS-Gesamtscores von EG zu VU bzw. NU.
- **Remission**: MADRS-Gesamtscore ≤10 zu VU bzw. NU.

Die Definitionen werden einheitlich für VU und NU angewendet; EG dient ausschließlich als Baseline für die Berechnung von Response/Remission.

Analytische Endpunkte:
Für alle Patient:innen mit MADRS zu EG und VU bzw. NU:

- Anteil (%) Responder zu VU und zu NU (inkl. 95 %-Konfidenzintervall).
- Anteil (%) Remitter zu VU und zu NU (jeweils 95 %-KI).
- Unterschied der gepaarten Response- bzw. Remissionsraten zwischen VU und NU (mit p-Wert und 95 %-Konfidenzintervall).

Statistisches Modell:

- **Primär:** rein deskriptiv
  N, n (%), 95 %-KI (z. B. Clopper–Pearson) für Response und Remission zu VU und NU.
- **Explorativ:** Einfacher gepaarter Test nach McNemar
  Prüfung, ob sich die Response/Remissions-Rate zwischen VU und NU unterscheidet.

# 10. Sekundäre Endpunkte D - Sicherheit und Suizidalität

Definition:

Sekundärer Endpunkt D beschreibt das Sicherheitsprofil der Esketamin-Induktion in der Induktions-Analysepopulation sowie Veränderungen suizidaler Symptome.

- **Sicherheit:** Häufigkeit und Art von behandlungsassoziierten unerwünschten Ereignissen (AEs) während der Induktionsphase.
- **Suizidalität:** Auftreten suizidalitätsbezogener AEs sowie Veränderung der suizidbezogenen Items (MADRS Item 10, PHQ-9 Item 9) über die Induktion.

Analytische Endpunkte:

**Sicherheit (AE-basiert, Induktionsphase):**

- Anteil (%) mit ≥1 AE, das als mit Esketamin in Zusammenhang stehend beurteilt wurde.
- Anteil (%) mit ≥1 schwerwiegendem AE (SAE) und Beschreibung dieser.
- Anteil (%) der Patient:innen, die die Induktion aufgrund eines AE abbrechen, sowie Häufigkeit der wichtigsten AE-Kategorien, die zum Abbruch führen.
- Anteil (%) mit mindestens einer dokumentierten Episode von Dissoziation.
- Anteil (%) mit mindestens einer dokumentierten Episode von starker Sedierung.
- Anteil (%) mit mindestens einem RR-bezogenen AE (Anstieg von ≥40 mmHg systolisch oder ≥25 mmHg diastolisch gegenüber dem jeweiligen Wert vor Behandlungsbeginn) während der Induktion.
- Anteil (%) mit mindestens einem sonstigen vordefinierten AE (z. B. Übelkeit, Erbrechen, Kopfschmerzen) während der Induktion.
- Anteil (%) an Behandlungssessions mit irgendeiner der o.g. UAW in Relation zur Gesamtzahl an Behandlungssessions.
- Anzahl und Beschreibung aller dokumentierten Ereignisse von vermutetem Missbrauch/Abusus, Diversion, Intoxikation oder Non-Adhärenz.

Alle Anteile werden als n (%) mit 95 %-Konfidenzintervallen berichtet.

**Suizidalität (AE-basiert und symptom-basiert):**

- Anteil (%) der Patient:innen mit mindestens einem AE, das als suizidale Ideation, Suizidversuch oder Selbstverletzung kodiert ist (während der Induktion), mit 95 %-Konfidenzintervall. Hierzu auch Anteil (%) an PatientInnen, die während der Therapie eine Erhöhung von MADRS item 10 oder PHQ-9 item 9 erlebt haben.
- Veränderung von MADRS Item 10 (suizidale Gedanken) von VU zu NU:
  - LSMean zu VU und NU, LSMean-Change VU → NU mit 95 %-Konfidenzintervall.
- Veränderung von PHQ-9 Item 9 (suizidale Gedanken) von Behandlungsbeginn zu Ende der Induktion:
  - LSMean vor Erstbehandlung (trt01) und bei Endpunkt (trt08), LSMean-Change mit 95 %-Konfidenzintervall.

# 11. Umgang mit fehlenden Daten

- Hauptanalysen: Mixed Models unter Missing at Random (MAR)-Annahme
- Sensitivitätsanalysen: Baseline-adjustiertes Modell, Multiple Imputation by chained equations (MICE), Delta-Adjustment (MNAR-Sensitivität)‑
- Sicherheitsanalysen: keine Imputation

# 12. Interpretation

- Signifikanzniveau für alle p Werte: α = 0,05 (zweiseitig)
- Keine Korrektur für multiples Testen in dieser SAP Version
  (spätere Module können eigene Adjustierungen enthalten)

Der primäre Endpunkt (Veränderung des MADRS-Gesamtscores VU → NU) wird konfirmatorisch ausgewertet. Alle weiteren Endpunkte (sekundäre und explorative Analysen, einschließlich MADRS-Faktoren, PHQ-9-Trajektorien, Response/Remission sowie Sicherheits- und Suizidalitätsanalysen) werden als explorativ betrachtet. Aufgrund des explorativen Charakters und der Vielzahl an Endpunkten erfolgt keine formale Adjustierung für multiples Testen. Für diese Analysen stehen Effektgrößen und 95 %-Konfidenzintervalle im Vordergrund; p-Werte werden als deskriptive Maßzahlen berichtet und nicht für scharfes Hypothesentesten interpretiert.

# 13. Software

Die Auswertungen erfolgen mit R oder einer äquivalenten validierten Statistiksoftware.
Versionen und verwendete Pakete werden im Analysereport dokumentiert.

# 14. Literaturverzeichnis

1.	Fedgchin M, Trivedi M, Daly EJ, et al. Efficacy and Safety of Fixed-Dose Esketamine Nasal Spray Combined With a New Oral Antidepressant in Treatment-Resistant Depression: Results of a Randomized, Double-Blind, Active-Controlled Study (TRANSFORM-1). *Int J Neuropsychopharmacol* 2019; **22**(10): 616–30.

2.	Borentain S, Gogate J, Williamson D, et al. Montgomery-Asberg Depression Rating Scale factors in treatment-resistant depression at onset of treatment: Derivation, replication, and change over time during treatment with esketamine. *Int J Methods Psychiatr Res* 2022; **31**(4): e1927.

3.	Batterham PJ, Calear AL, Farrer L, Gulliver A, Kurz E. Efficacy of a Transdiagnostic Self-Help Internet Intervention for Reducing Depression, Anxiety, and Suicidal Ideation in Adults: Randomized Controlled Trial. *J Med Internet Res* 2021; **23**(1): e22698.
